# The COVID-19 Schools Infection Survey in England: Protocol and Participation Profile for a Prospective Observational Cohort Study

**DOI:** 10.2196/34075

**Published:** 2022-11-10

**Authors:** Katherine E Halliday, Patrick Nguipdop-Djomo, William E Oswald, Joanna Sturgess, Elizabeth Allen, Neisha Sundaram, Georgina Ireland, John Poh, Samreen Ijaz, Justin Shute, Ian Diamond, Emma Rourke, Fiona Dawe, Alison Judd, Taane Clark, W John Edmunds, Chris Bonell, Punam Mangtani, Shamez N Ladhani, Sinéad M Langan, James Hargreaves, COVID-19 Schools Infection Survey Study Group

**Affiliations:** 1 Department of Disease Control Faculty of Infectious and Tropical Diseases London School of Hygiene & Tropical Medicine London United Kingdom; 2 Department of Infectious Disease Epidemiology Faculty of Epidemiology and Population Health London School of Hygiene & Tropical Medicine London United Kingdom; 3 Department of Medical Statistics Faculty of Epidemiology and Population Health London School of Hygiene & Tropical Medicine London United Kingdom; 4 Department of Global Health and Development Faculty of Public Health and Policy London School of Hygiene & Tropical Medicine London United Kingdom; 5 UK Health Security Agency London United Kingdom; 6 Office for National Statistics Government Buildings Newport United Kingdom; 7 Department of Infection Biology Faculty of Infectious and Tropical Diseases London School of Hygiene & Tropical Medicine London United Kingdom; 8 Department of Public Health, Environments and Society Faculty of Public Health and Policy London School of Hygiene & Tropical Medicine London United Kingdom; 9 Department of Non-communicable Disease Epidemiology Faculty of Epidemiology and Population Health London School of Hygiene & Tropical Medicine London United Kingdom; 10 See Acknowledgments

**Keywords:** COVID-19, SARS-CoV-2, school-based, epidemiology, infection control

## Abstract

**Background:**

One of the most debated questions in the COVID-19 pandemic has been the role of schools in SARS-CoV-2 transmission. The COVID-19 Schools Infection Survey (SIS) aims to provide much-needed evidence addressing this issue.

**Objective:**

We present the study protocol and participation profile for the SIS study, aimed at assessing the role of schools in SARS-CoV-2 infection and transmission within school settings, and investigating how transmission within and from schools could be mitigated through the implementation of school COVID-19 control measures.

**Methods:**

SIS was a multisite, prospective, observational cohort study conducted in a stratified random sample of primary and secondary schools in selected local authorities in England. A total of 6 biobehavioral surveys were planned among participating students and staff during the 2020-2021 academic year, between November 2020 and July 2021. Key measurements were SARS-CoV-2 virus prevalence, assessed by nasal swab polymerase chain reaction; anti-SARS-CoV-2 (nucleocapsid protein) antibody prevalence and conversion, assessed in finger-prick blood for staff and oral fluid for students; student and staff school attendance rates; feasibility and acceptability of school-level implementation of SARS-CoV-2 control measures; and investigation of selected school outbreaks. The study was approved by the United Kingdom Health Security Agency Research Support and Governance Office (NR0237) and London School of Hygiene & Tropical Medicine Ethics Review Committee (reference 22657).

**Results:**

Data collection and laboratory analyses were completed by September 2021. A total of 22,585 individuals—1891 staff and 4654 students from 59 primary schools and 5852 staff and 10,188 students from 97 secondary schools—participated in at least one survey. Across all survey rounds, staff and student participation rates were 45.2% and 16.4%, respectively, in primary schools and 30% and 15.2%, respectively, in secondary schools. Although primary student participation increased over time, and secondary student participation remained reasonably consistent, staff participation declined across rounds, especially for secondary school staff (3165/7583, 41.7% in round 1 and 2290/10,374, 22.1% in round 6). Although staff participation overall was generally reflective of the eligible staff population, student participation was higher in schools with low absenteeism, a lower proportion of students eligible for free school meals, and from schools in the least deprived locations (in primary schools, 446/4654, 9.6% of participating students were from schools in the least deprived quintile compared with 1262/22,225, 5.7% of eligible students).

**Conclusions:**

We outline the study design, methods, and participation, and reflect on the strengths of the SIS study as well as the practical challenges encountered and the strategies implemented to address these challenges. The SIS study, by measuring current and incident infection over time, alongside the implementation of control measures in schools across a range of settings in England, aims to inform national guidance and public health policy for educational settings.

**International Registered Report Identifier (IRRID):**

RR1-10.2196/34075

## Introduction

### Context and Rationale

The novel coronavirus *SARS-CoV-2* outbreak was declared a global pandemic by the World Health Organization on March 11, 2020 [1,2]. By this date, *lockdowns*, including school closures, had begun to be implemented worldwide [3]. Early evidence indicated that children aged <18 years were significantly less likely to develop severe disease or die than adults [4], but asymptomatic cases could also contribute to disease spread [5]. Evidence from previous influenza outbreaks had identified children as the main drivers of infection, with school closures having a positive impact on infection control in the community [6]. Early in the pandemic, however, the extent of asymptomatic infection and the role of children and school environments in the transmission and control of SARS-CoV-2 were unclear [3,7-9].

Evidence indicated that on March 11, 2020, a total of 29 countries had implemented national school closures, and by March 18, 2020, this had increased to 107 countries [10]. In England, schools were closed for in-person teaching from March 23, 2020, to all but children of key workers and vulnerable children [11]. However, school closures are linked to detrimental educational, social, mental health, and well-being impacts on children [12,13]. Negative economic and well-being effects are also seen in families, with inequity in such impacts seen across income backgrounds and ethnicity and in single-parent households [14,15]. Consequently, school closures have been considered a measure of last resort with the policy intent of limiting closures to a minimum. Therefore, there was an urgent need to understand transmission within schools and the potential risk of transmission to and from communities [16], as well as ways of minimizing these risks when schools were open [17-19].

Research and surveillance activities to assess SARS-CoV-2 transmission in communities, hospitals, and care homes in England were initiated during the lockdown in spring 2020 [20,21,22]. However, the investigation of SARS-CoV-2 transmission in schools in the United Kingdom was primarily limited to modeling studies conducted between March 2020 and June 2020 [23-25]. International outbreak investigations early in the pandemic suggested that although schools are a potential source of infection, for the most part, few infection clusters were linked to schools, attack rates were reported to be low, and there was evidence of reduced susceptibility to infection in younger children [26]. Some exceptions were noted; for example, school outbreak studies in France and Israel were considered linked to poor infection control practices [27-29].

As the first wave of the pandemic eased in England, schools began a phased reopening in June 2020, limited initially to academic years 1 (ages 5-6 years) and 6 (ages 10-11 years) in primary schools and years 10 (ages 14-15 years) and 12 (ages 16-17 years) in secondary schools. Government guidance was produced for schools on implementing social distancing and infection control measures, including limiting class sizes, grouping children in *bubbles* and limiting contact between bubbles; hand washing and hygiene; and requiring those with a positive SARS-CoV-2 test result, alongside their contacts, to remain at home. This partial reopening was successful in that there were very low rates of infection and outbreaks during the 6 weeks of partial reopening of schools [30,31]. From September 2020, all students were invited to return to full-time in-person teaching in England. In addition to concerns about the impact of increased transmission exposure on students, staff, and their families, there were additional challenges faced by staff in implementing, and both staff and students in following, school preventive measures [10,32].

Despite an increase in the number of studies investigating SARS-CoV-2 transmission and its impact on school-age children worldwide [33,34] and in England specifically [20,30,31,35], there remained a limited number of studies following large cohorts of staff and students assessing both point prevalence and antibody conversion, school-level preventive measures, and changes in behaviors and perceptions during periods of high and low community infection rates. The Schools Infection Survey (SIS), a collaboration among the London School of Hygiene & Tropical Medicine, UK Health Security Agency (UKHSA), and Office for National Statistics (ONS), was therefore rapidly commissioned by the UK Department of Health and Social Care to provide critical data from the 2020-2021 academic year to fill this gap.

### Aims and Objectives

The aim of the COVID-19 SIS was to assess the role of schools in SARS-CoV-2 infection and transmission within school settings and investigate how transmission within and from schools could be minimized by exploring the implementation and feasibility of school COVID-19 prevention and control measures. SIS specific objectives, within selected primary and secondary schools, were as follows:

1. Estimate SARS-CoV-2 antibody prevalence and incidence based on antibody conversion among students and staff, measured at termly intervals during the school year.
2. Measure the prevalence of current SARS-CoV-2 infection among students and staff, measured at half-termly intervals during the school year.
3. Monitor attendance rates and the proportion of and reasons for full or partial school closure.
4. Assess the feasibility; acceptability; and staff, student, and parent experience of school implementation of SARS-CoV-2 control measures and the factors affecting their implementation.
5. Conduct investigations of selected outbreaks in schools to determine the risk of transmission within and between classes and schools and among students, staff, and other household members.

In addition to the 5 primary objectives mentioned, we planned to investigate contact patterns and evaluate individual-, school-, and community-level risk factors.

In this paper, we present the study design and protocol, recruitment, and participant profile across the 2020-2021 academic year and discuss SIS strengths, challenges, and adaptations during the study period—between November 2020 and July 2021.

## Methods

### Study Design

**Figure 1:**
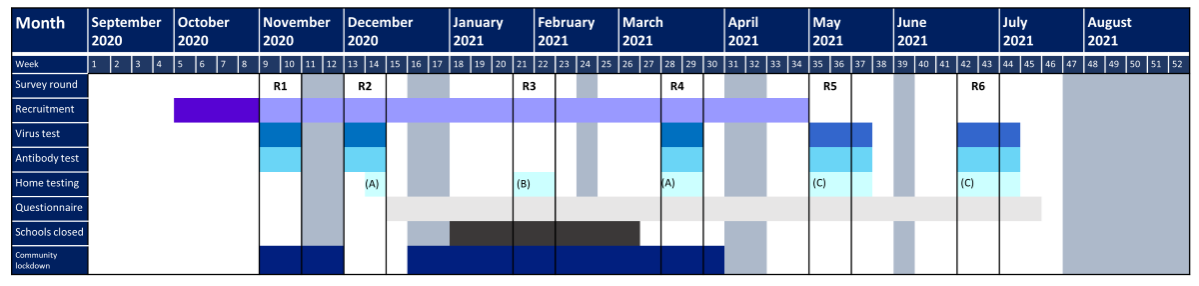
The Schools Infection Survey study design and time line. Grey columns indicate school holiday periods. Dark purple indicates initial school and participant recruitment period and light purple indicates period of rolling recruitment for schools and participants. (A) indicates home-testing kits (antibody tests and viral swabs) for any participants who were not in school on the day of the school surveys, (B) indicates home-testing kits (antibody tests and viral swabs) for those who enrolled by January 28, 2021, and did not have an antibody result from either round 1 or 2 surveys. (C) indicates home-testing kits (antibody tests only) for any participants who were not in school on the day of the school surveys. R: round.

SIS is a cohort study in which biological samples for virus and antibody tests and questionnaire data were collected from staff and students at regular intervals throughout the school year (Figure 1), with antibody prevalence and conversion, as well as viral prevalence, at points in the academic year as key outcome measures. Electronic questionnaires were used to collect data on the risk factors for infection and additional indicators, such as prior positive tests for SARS-CoV-2. We also obtained school attendance records; conducted implementation research to assess the implementation of preventive measures within schools as well as perceptions of their feasibility, acceptability, and broader impact through surveys and semistructured interviews; and conducted detailed investigations in selected schools where there were suspected outbreaks.

### Study Setting and Population

SIS involved students and staff attending government primary and secondary schools in selected upper-tier local authorities (LAs) in England during the 2020-2021 academic year. The exclusion criteria for schools included special schools, student referral units and further education colleges (owing to the recognition that infection control issues and procedures were in many cases likely to differ in these settings), independent schools (for logistical reasons), and schools where other school-based COVID-19 studies were being conducted. Eligible participants included primary and secondary school students and staff for whom informed consent was provided. Year 11 students were excluded to minimize disruption of public examinations at the end of the academic year.

The design and implementation of the SIS study was informed by the prior COVID-19 Surveillance in School KIDs (sKIDs) study, which was initiated as schools partially reopened in June 2020 and involved weekly nasal swabbing and blood sampling among staff and students [30]. The sKIDs study also included an accompanying social science study of feasibility, challenges, and facilitators associated with the implementation of preventive measures at schools as well as the acceptability of biological testing in schools [36]. Formative qualitative research in the form of interviews and focus group discussions undertaken in the sKIDs study, with head teachers, parents, and students, guided the development of many of the research questions in SIS.

### Sampling

A multistage stratified sampling scheme was used. The first level of sampling was at the LA level. To study transmission, we aimed to oversample schools in parts of England where the risk of SARS-CoV-2 infection was higher at the beginning of the 2020-2021 school year. All 149 LAs in England were stratified according to the population rate of confirmed SARS-CoV-2 infection per 100,000 population from *pillar 2* testing—a centralized system of community-based swab testing—in the week, September 2, 2020, to September 8, 2020. Group 1 constituted the top 20% of LAs when ranked by transmission, and group 2 comprised LAs in the lower 80%. A total of 15 LAs were selected using simple random sampling, 10 from group 1 and 5 from group 2.

The sampling frame subsequently included all primary and secondary schools within the 15 selected LAs [37]. Sample allocation also prioritized secondary schools, as SARS-CoV-2 transmission appeared to be higher among older children [38]. Primary and secondary schools were sampled separately, with the aim of a sample ratio of 1:2 and approximately 70% (35 primary and 70 secondary) of schools from group 1 and 30% (15 primary and 30 secondary) from group 2. Because of the differing LA sizes and school numbers per LA, inverse probability weighting was used to distribute the sample more evenly across the LAs. For pragmatic reasons, the maximum number of academic trust–managed schools was capped at 4 per LA to allow efficient engagement.

In primary schools, where the average school population was 280 students, enrollment was offered to all staff and students. In secondary schools, which are larger, with an average school population of 990 students, for logistical reasons, we initially randomly selected 2 consecutive year groups (eg, years 7-8, 9-10, and 12-13; approximately 250 students) per school, in addition to offering enrollment to all staff [39].

Overall, 2 modifications were made to the sampling strategy following the commencement of SIS. First, in certain LAs where school enrollment in SIS was low after round 2, additional schools were sampled and invited to participate to increase the representativeness of all LAs sampled. Second, to increase student recruitment in secondary schools, schools were encouraged to open up enrollment to the remaining school years (except year 11) from January 2021.

### Sample Size Considerations

The overall target sample size was principally influenced by pragmatic concerns, as it was constrained by antibody and virus testing capacity, with an estimated 40,000 tests deemed feasible for processing in laboratories per round. We assumed a response and follow-up rate of 60% among students and up to 90% among the staff. Schools were oversampled (approximately 250 schools) to compensate for school-level refusals and achieve participation of 150 schools.

We estimated cumulative incidence based on antibody conversion and its precision, assuming approximately 10% of students and staff would have a positive antibody test at enrollment, an average weekly incidence of 1 infection per 1000 individuals, with limited antibody reversion, and assuming a design effect of 2.3 for antibody testing to account for clustering because of sampling entire schools or school year groups. The design effect of 2.3 assumes an average prevalence of 10% antibody at enrollment and a between-school SD of 2.5% (ie, 95% of schools are between 5% and 15%), with 150 to 200 students enrolled per school. On the basis of the sample sizes, Table 1 presents the cumulative incidence of antibody conversion over different follow-up periods with statistical precision for each group at the 95% confidence level.

**Table 1:** Sample size required to detect certain antibody conversion rates with 95% CIs at different follow-up periods.

Individuals included and antibody conversion rate		4 weeks between follow-up	8 weeks between follow-up	12 weeks between follow-up
**Secondary staff, approximate n=10,620**				
	Antibody conversion, % (95% CI)	0.4 (0.2-0.6)	0.8 (0.5-1.1)	1.2 (0.9-1.5)
	Converting, n (95% CI)	42 (21-64)	85 (53-117)	127 (96-159)
**Secondary students, approximate n=20,400**				
	Antibody conversion, % (95% CI)	0.4 (0.3-0.5)	0.8 (0.6-1.0)	1.2 (1.0-1.4)
	Converting, n (95% CI)	82 (41-122)	163 (102-224)	245 (184-306)
**Primary staff, approximate n=1440**				
	Antibody conversion, % (95% CI)	0.4 (0.0-0.9)	0.8 (0.1-1.5)	1.2 (0.3-2.1)
	Converting, n (95% CI)	6 (3-9)	12 (7-16)	17 (13-22)
**Primary students, approximate n=8460**				
	Antibody conversion, % (95% CI)	0.4 (0.2-0.6)	0.8 (0.5-1.1)	1.2 (0.8-1.6)
	Converting, n (95% CI)	34 (17-51)	68 (42-93)	102 (76-127)

### Engagement and Recruitment

Initial engagement and recruitment processes were carried out via email with the support of study engagement officers directly liaising with schools. We used a cascade approach: emailing a letter and information sheet to union officials, LA heads of education, directors of public health, and academic trust leaders. Head teachers at all 250 schools in the original sampling frame were contacted via email, with a letter detailing the study objectives and procedures. Head teachers were invited to enroll their school via a weblink and requested to email an invitation letter, information sheet, and registration link to all staff and parents or guardians in primary schools and all staff as well as parents from the 2 prespecified year groups in secondary schools. The schools were requested to email students aged ≥16 years (eg, in years 12 and 13). All eligible participants and parents or guardians were provided with instructions on completing a web-based informed consent form and enrollment questionnaire. Informed consent was obtained from staff, students aged ≥16 years, and parents or guardians of children aged 4 to 15 years via a secure web-based portal before enrollment. Verbal assent was obtained before biological sample collection at the school.

Because of the slow initial enrollment, several modifications were made to the engagement and recruitment strategies. First, a transition was made from a closed to an open cohort with rolling recruitment until round 5 in May 2021. Second, we developed a suite of paper-based communication materials to increase the accessibility of information, although enrollment was still digital via an email link, and several school-based internet-based forums were held to engage parents and students more directly. Finally, from 2021, participating schools were also provided with compensation (for staff time use to support the study and not linked to any recruitment target in the school or other contingency).

### Time Line

School recruitment began on October 12, 2020, and all data collection and laboratory analyses were completed by September 2021. In total, 6 rounds of data collection were planned for the school year. The first round of surveys was conducted between November 3, 2020, and November 20, 2020, and the second round between November 30, 2020, and December 11, 2020, with the third, fourth, fifth, and sixth rounds planned for January 9, 2021, March 15, 2021, May 5, 2021, and June 14, 2021, respectively (Figure 1). Because of the second national lockdown and partial school closures starting on January 5, 2021, the time line and format of the round 3 surveys were altered, and a round of home testing for antibodies was implemented between January 29, 2021, and February 9, 2021.

### Data Collection

#### Virus and Antibody Samples

Research teams visited schools on preagreed days with preprepared barcoded sample kits to collect virus and antibody samples from enrolled students and staff. Testing conducted in schools was not intended to replace routine national testing for those experiencing symptoms, and any staff or students experiencing symptoms were advised to visit routine services and were not expected to attend school on testing days. SARS-CoV-2 infection testing was conducted via nasal swabs for viral detection using reverse transcriptase–polymerase chain reaction (RT-PCR). For staff and secondary school participants, nasal swabs were self-administered and obtained from primary school children by nurses in the research teams. SARS-CoV-2 antibody testing for students was carried out on an oral fluid sample [40], in which children collected transudates from the gingival crevice using an Oracol foam swab (Malvern Medical Developments Ltd), thus limiting the contribution of salivary gland secretions. Oral fluid sampling was used for students as it is less invasive than blood sampling and is painless; therefore, it is more likely to encourage participation by students. SARS-CoV-2 antibody testing for staff was performed on a self-collected finger-prick capillary blood sample.

The home-based testing approach supplemented the in-school surveys from round 2 with virus and antibody tests sent to enrolled individuals unavailable at school on the survey day, who could be reached by telephone. Round 3 was implemented exclusively through home testing, during which any participant who had enrolled by the end of January 2021 but had not yet provided a sample for antibody testing (including participants in schools that had opted out of round 1 or round 2 testing) were contacted and sent a home-testing kit to obtain baseline measures for future antibody conversion estimates. From round 4 (March 2021) onward, home-test antibody kits were sent to individuals who were unavailable at school on the survey day.

Nasal swabs were sent to a national testing center for RT-PCR assay on an Applied Biosystems 7500 FAST system targeting a conserved region of the open reading frame (ORF1ab) gene, as well as the N and S genes of SARS-CoV-2 [30]. Oral fluid swabs were sent to UKHSA Colindale for detection of antibodies against the SARS-CoV-2 nucleoprotein using an in-house immunoglobulin G-capture–based enzyme immunoassay [40], and the staff capillary blood samples were tested with a validated commercial immunoassay for total antibodies against the SARS-CoV-2 nucleoprotein antigen (Roche cobas Elecsys Anti-SARS-CoV-2 assay; Roche Diagnostics). Positive RT-PCR test results were communicated to participants or parents via telephone within 48 hours of the laboratory results. The National Health Service Test and Trace was also informed of any positive results in line with current regulations, and participants were advised to self-isolate according to national guidelines. Negative viral RT-PCR test results were communicated via a secure participant web-based portal. Antibody results, whether positive or negative, were also communicated via a secure participant web-based portal.

#### Questionnaires

School-level information, including student and staff head counts, guidance received by the school on prevention, and implementation and feasibility of school infection prevention and control measures, was collected via headteacher questionnaires. Participants or their parents or guardians received a brief web-based questionnaire at enrollment, requesting information on demographic characteristics, postcode, household size, school year group for students, and role for staff. Following the collection of samples at each round, participants were requested to complete additional web-based questionnaires, including questions on household composition, medical history including previous COVID-19 infection, recent symptoms, contacts within and outside of school, activities, travel, mental well-being, and COVID-19 vaccination sentiment and uptake. Further questions covered the adoption of and consistency with recommended prevention and control measures in schools. The questionnaires provided critical information for addressing all 5 study objectives and were completed through a secure participant web-based portal and were linked to samples through a unique identifier. Refer to Multimedia Appendix 1 for the questionnaires used during SIS. Staff and students aged ≥16 years completed the questionnaire themselves, and parents or guardians were requested to complete the questionnaire on behalf of, and in consultation with, their children, as appropriate. An extensive questionnaire was completed at the first visit, with subsequent updates of relevant information in each round of testing, using a shorter *follow-up* questionnaire.

#### Attendance Data

School-level absence data for participating schools were obtained from the Department for Education through the Educational Setting Status service for the 2020-2021 academic year to address study objective 3. These daily data are disaggregated by staff and students and include whether the school setting is open and the reason for closure if applicable, as well as daily student and staff absences in total and those related to COVID-19–related reasons (eg, suspected case, confirmed case, or potential contact with a case including self-isolation). In addition, questionnaires administered following the surveys contained questions on the number of days absent in the preceding 4 weeks, whether COVID-19–related, and if so, the COVID-19–related reason for absence.

#### Other Contextual and Linked Data Sources

In addition to the data collected by the study directly, contextual-level information available from other sources about participating LAs, schools, and participants was obtained, including open access school-level data such as location, school type, percentage of students eligible for free school meals (FSMs), performance, workforce, from the Department for Education [37,41] and postcode-level 2019 deprivation data from the Ministry of Housing, Communities & Local Government [42]. In addition, data on case rates from pillar 2 testing [43] and where possible, relevant estimates of community virus and antibody prevalence rates from the COVID-19 Infection Survey (CIS) were used for comparison [44]. At the school and individual levels, consent from participants was sought to link data obtained through this survey with other survey and administrative data held by the ONS, which included (1) test and trace regarding COVID-19 tests and results and (2) the National Immunization Management Service providing information on participants’ COVID-19 vaccination status.

#### Qualitative Data

A nested longitudinal qualitative study was undertaken in a subsample of schools with key stakeholder groups (headteachers, teachers, parents or guardians, and students) to better understand the experience at schools during the pandemic; implementation, feasibility, and acceptability of school control measures; and impact of COVID-19 and mental well-being (study objective 4). Among schools indicating willingness to participate in the qualitative research, a minimum of 6 schools were purposively selected based on the following criteria: school type, local deprivation, and responses regarding the implementation of school measures in the head teacher questionnaire.

Semistructured interviews were conducted with head teachers, teachers, and parents or guardians at primary schools and with head teachers, teachers, parents or guardians, and students at secondary schools. Information about the study and consent forms were circulated to the participants in advance. All interviews were conducted via telephone and audio recorded with participant permission following the provision of informed consent. Repeat interviews were conducted with the same participants at another time point during the school year. A total of 74 interviews across 4 primary and 4 secondary schools were completed in rounds 1 and 2 of the nested qualitative study. In all, 43 interviews were conducted in round 1 between February 2021 and April 2021. In round 2, repeat interviews were conducted with 31 participants from round 1 between June 2021 and July 2021. The results of this nested qualitative study will be presented in future publications.

#### Outbreak Investigation

As part of its commitment to public health management of COVID-19 in institutional settings, UKHSA coordinated risk assessments and investigations in selected school *bubbles* with one or more positive cases, including wider testing among staff, students, and their households, as identified by the risk assessment. The outbreak investigation protocol, exploring study objective 5, used a home-testing approach based on SARS-CoV-2 RT-PCR testing results within SIS and early alerts of other SARS-CoV-2 infections notified by schools. Data from the SIS outbreak investigations have been combined with data from outbreak investigations conducted as part of the sKIDs study in primary schools and the sKIDs PLUS study in secondary schools [30,45].

### Data Management and Analysis

#### Data Management

Data were collected via a secure web-based portal and linked by ONS. Participant ID and deidentified information were linked to the school by the school’s unique reference number. The results of the nasal swabs and antibody tests were linked to participants’ survey records using a barcoded ID. Deidentified data sets were made available to authorized investigators to be analyzed in the ONS Secure Research Service. The interview data were transcribed verbatim from audio recordings, with identifiers removed and enhanced with notes taken during the interview. Anonymized transcripts and notes are held on the London School of Hygiene & Tropical Medicine secure servers and managed and analyzed using MAXQDA (version 12; VERBI GmbH).

#### Results Reporting and Dissemination

Descriptive results were rapidly reported in publicly available bulletins published by ONS after each survey round to inform national policy discussions [46]. These include PCR-based viral test positivity and antibody test positivity, both of which are unadjusted for diagnostic test performance. The estimates were weighted to be representative of the relevant populations in the sampled LAs. The weighted test positivity for SARS-CoV-2 in each LA and time point was presented by the key population groups tested: primary school students and staff and secondary school students and staff.

A range of analyses addressing the study objectives will be presented in subsequent papers, including longitudinal analyses of antibody and infection prevalence, accounting for diagnostic test performance to address study objectives 1 and 2, and multilevel regression modeling to assess risk factors for infection and antibody prevalence and antibody conversion from negative to positive at the individual, school, and community levels. The correlation between school-level infection and COVID-19–related absence will be examined to address objective 3.

Objective 4, examining the implementation of preventive measures and staff well-being, will be addressed through quantitative analysis of the implementation of measures and associated challenges reported in the head teacher questionnaire and adherence to measures and teacher burnout reported in the staff questionnaire. Furthermore, qualitative analyses of the fidelity, feasibility, and acceptability of school implementation of COVID-19 control measures and their impact on well-being will use narrative data from interviews with head teachers, teachers, parents, and students. The general theory of implementation will be used as a theoretical framework to guide the design, analysis, and interpretation of findings [47]. Thematic analysis will be conducted to address the study objectives using a combination of both deductive and inductive coding approaches.

Objective 5, investigation of secondary attack rates and outbreaks, will be analyzed using mathematical modeling as well as through the analysis of school bubbles and their household contacts [45].

#### Current Analyses of Recruited Study Population

In this paper, we present a description of enrollment and the recruited population participating in SIS during the 2020-2021 academic year. School and individual participation are described in rounds 1 to 6. Enrolled schools are those, which submitted the school consent form and enrollment questionnaire before the first day of the survey round. The participating schools are schools visited by the survey team during that specific round. The eligible population in each round is estimated from the total staff census in participating primary and secondary schools, the total student census in participating primary schools, and all students in the 2 selected year groups in participating secondary schools. For schools that expanded enrollment to additional school years in January 2021, eligible students included all students (except year 11) during rounds 4 to 6. Enrolled individuals are defined as those who have submitted the consent form and enrollment questionnaire, and participating individuals are those who were present on the day the research team visited the school and had at least one sample taken.

Descriptive analyses of school-level characteristics of the sampled, eligible, and participating schools and participants were performed using unweighted frequencies and proportions for categorical variables. Individual-level sociodemographic characteristics are also described for those participating in any survey round. Data analyses were performed using Stata (version 16.0; StataCorp).

### Ethics Approval

The study was approved by the UKHSA Research Support and Governance Office (NR0237) and London School of Hygiene & Tropical Medicine Ethics Review Committee (reference 22657). Electronic informed consent was obtained from staff, students aged ≥16 years, and parents or guardians of children aged 4 to 15 years via a secure web-based portal before enrollment. Verbal consent or assent was obtained before sample collection at the school. Informed consent was obtained before interviews. Telephone helplines and responses to frequently asked questions on a weblink were available. Schools, staff, and parents or guardians of participating students and the students themselves were free to withdraw consent at any time.

## Results

Here, we provide a description of recruitment and participation by study round, as well as the characteristics of schools and individuals participating in the SIS study.

### LA Selection

**Figure 2:**
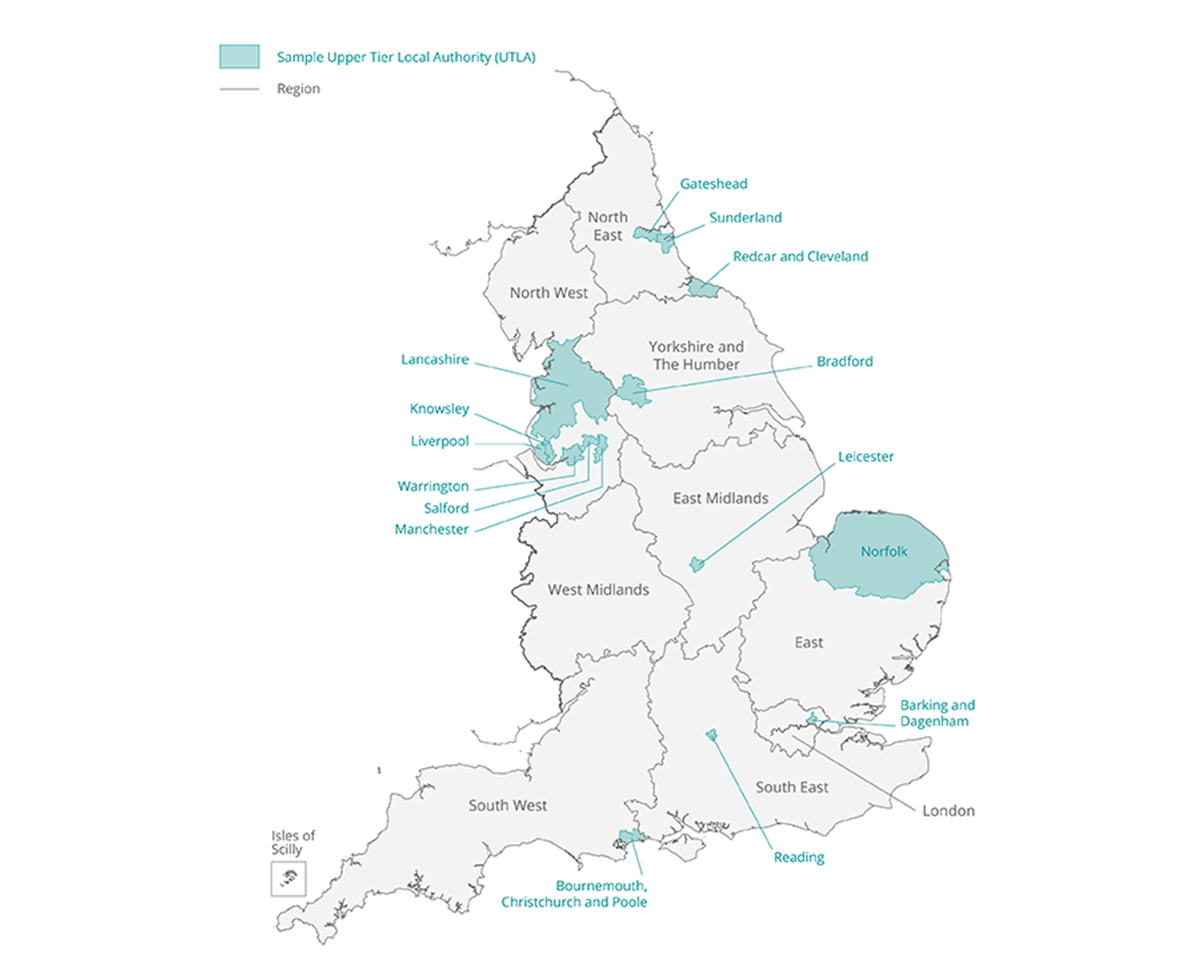
Map of local authority areas participating in the Schools Infection Survey study. UTLA: Upper Tier Local Authority.

In total, 10 of the LAs classified as in the top 20% of transmission (based on pillar 2 data) and 5 of the LAs classified as in the lower 80% of transmission were randomly selected. The median LA-level case rate from pillar 2 testing, the week of September 2, 2020, to September 8, 2020, was 121.9 per 100,000 population for LAs classified as *high transmission* and 18.9 per 100,000 population for LAs classified as *low transmission*. These LA-level case rates provided a snapshot of regional transmission at the start of the school year, which subsequently changed substantially between September and December 2020, rendering this distinction irrelevant. The selected LAs are located in 8 of the 9 regions in England, with 6 LAs located in the northwest region, 3 LAs in the northeast region, and 1 LA in each of the other 6 regions (Figure 2).

### School Enrollment, Participation, and Characteristics

**Figure 3:**
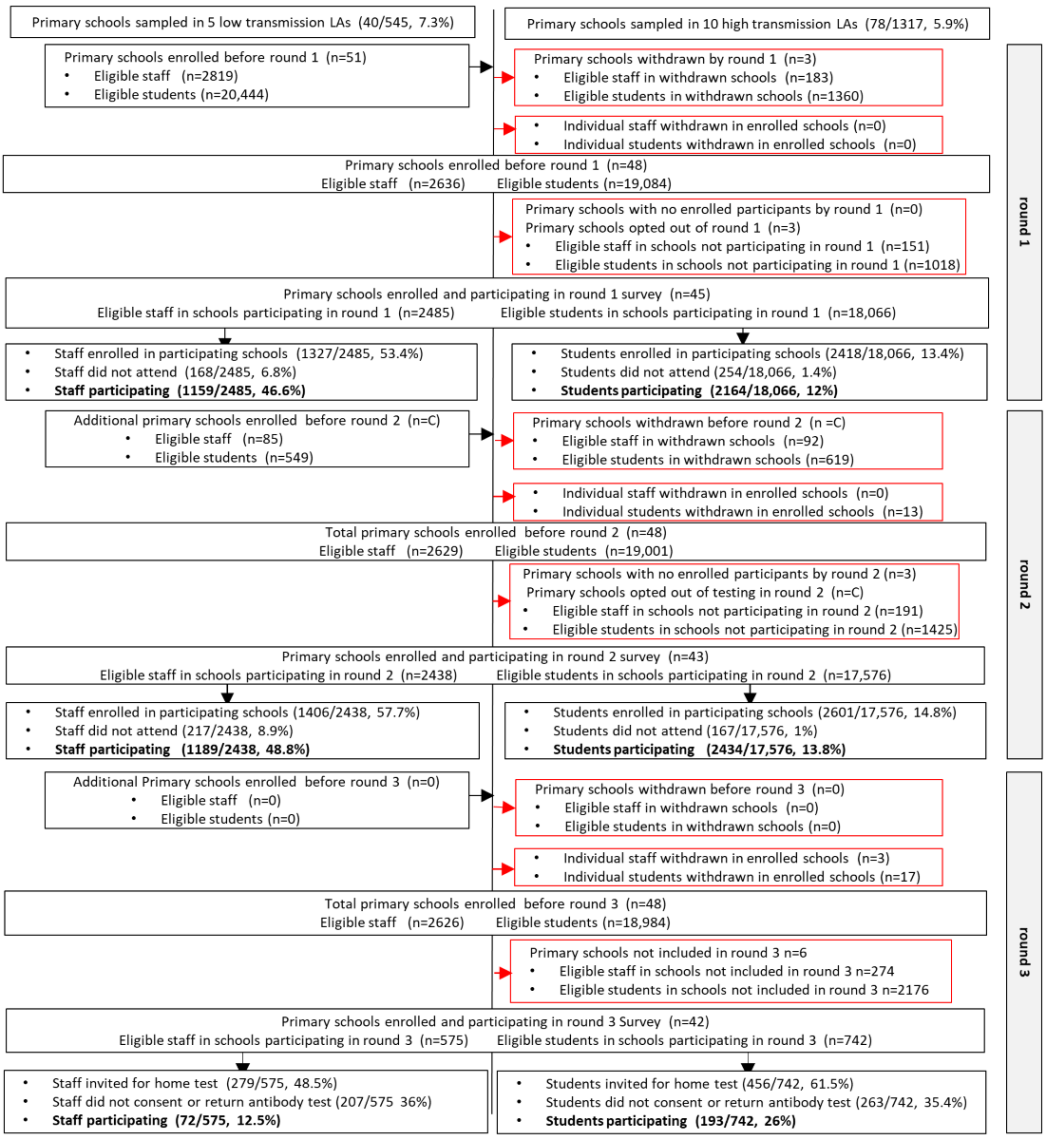
The Schools Infection Survey recruitment and participation profile for primary schools during rounds 1 to 3. Suppressed numbers are indicated by C Percentage of eligible refers to eligible participants within schools enrolled and participating in that round. LA: local authority.

**Figure 4:**
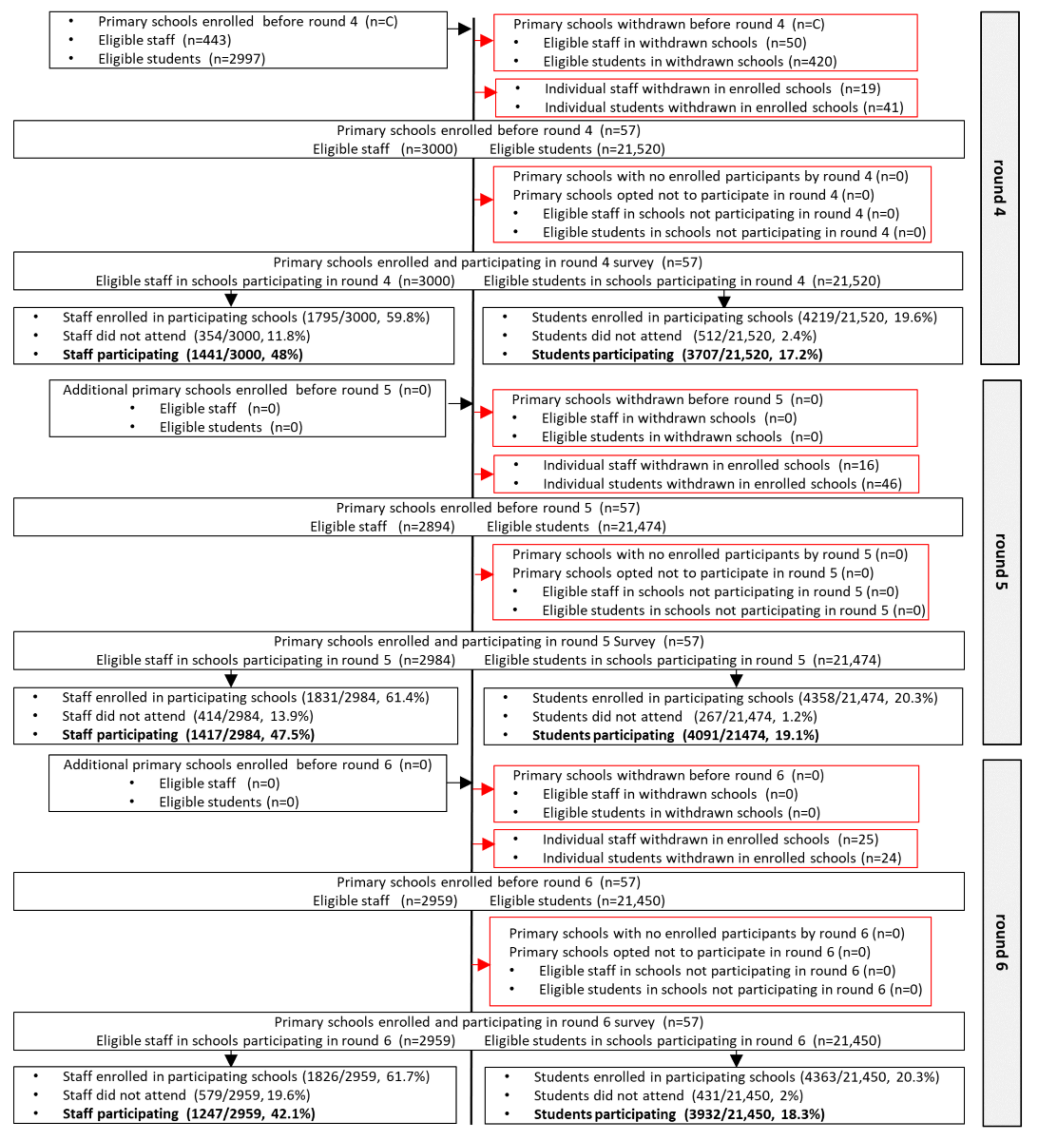
The Schools Infection Survey recruitment and participation profile for primary schools during rounds 4 to 6. Suppressed numbers are indicated by C Percentage of eligible refers to eligible participants within schools enrolled and participating in that round. LA: local authority.

**Figure 5:**
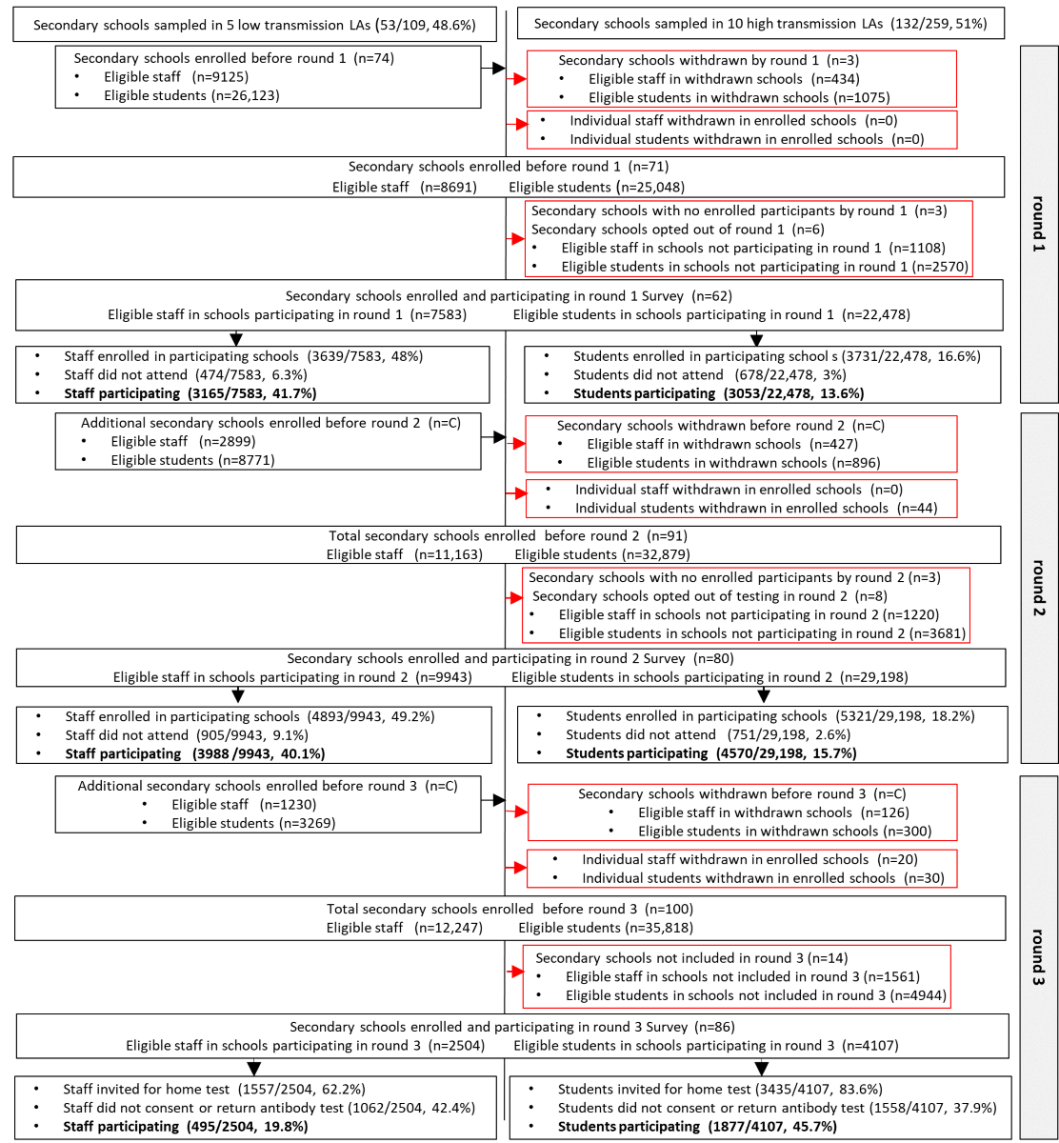
The Schools Infection Survey recruitment and participation profile for secondary schools during rounds 1 to 3. Suppressed numbers are indicated by C. Percentage of eligible refers to eligible participants within schools enrolled and participating in that round. In rounds 1 to 3, eligible secondary students refers to the 2 selected eligible classes. From January 2021, secondary schools were invited to open enrollment up to the whole school (excluding year 11), leading to an increase in eligible students in rounds 4 to 6. LA: local authority.

**Figure 6:**
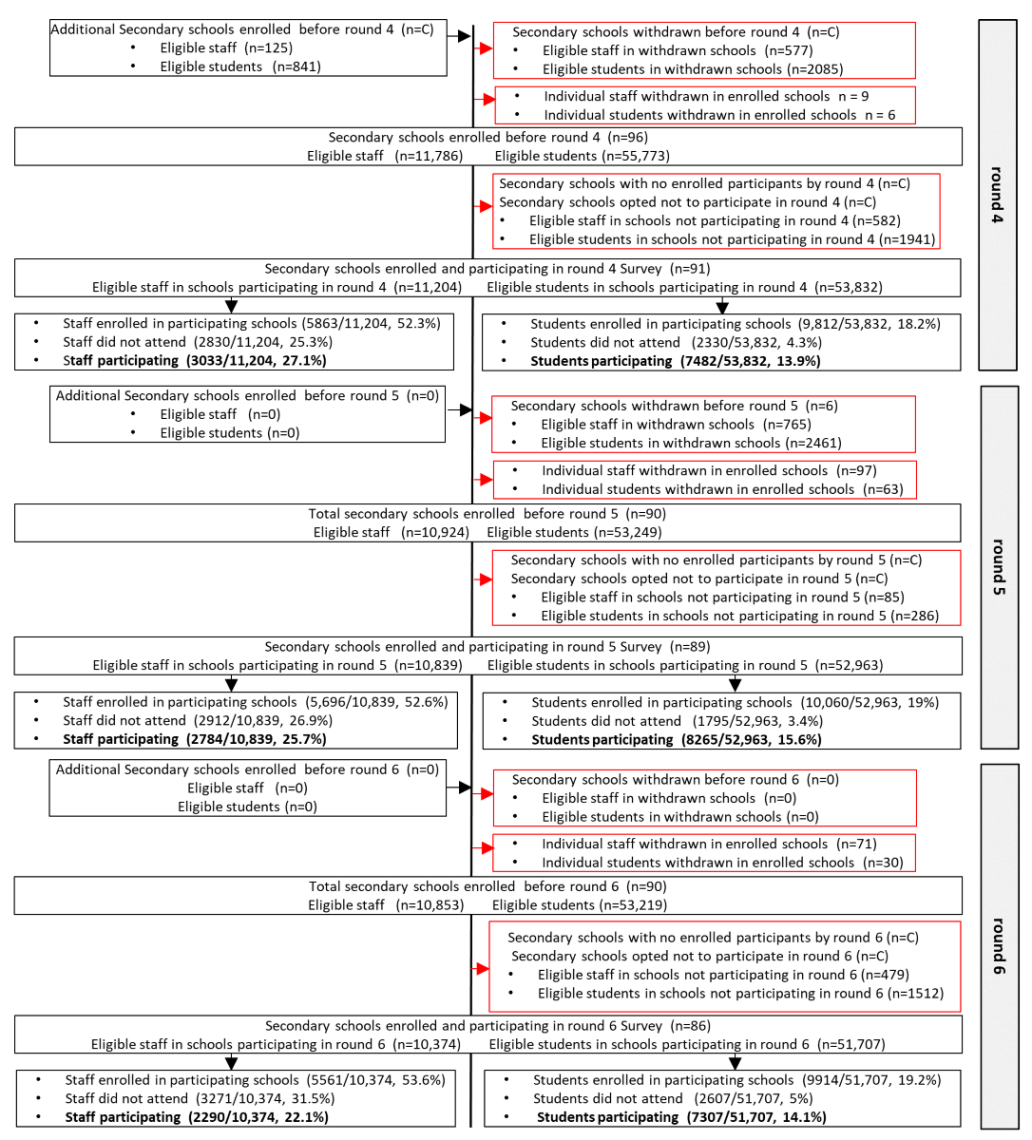
The Schools Infection Survey recruitment and participation profile for secondary schools during rounds 4 to 6. Suppressed numbers are indicated by C. Percentage of eligible refers to eligible participants within schools enrolled and participating in that round. In rounds 1 to 3, eligible secondary students refers to the 2 selected eligible classes. From January 2021, secondary schools were invited to open enrollment up to the whole school (excluding year 11), leading to an increase in eligible students in rounds 4 to 6. LA: local authority.

**Figure 7:**
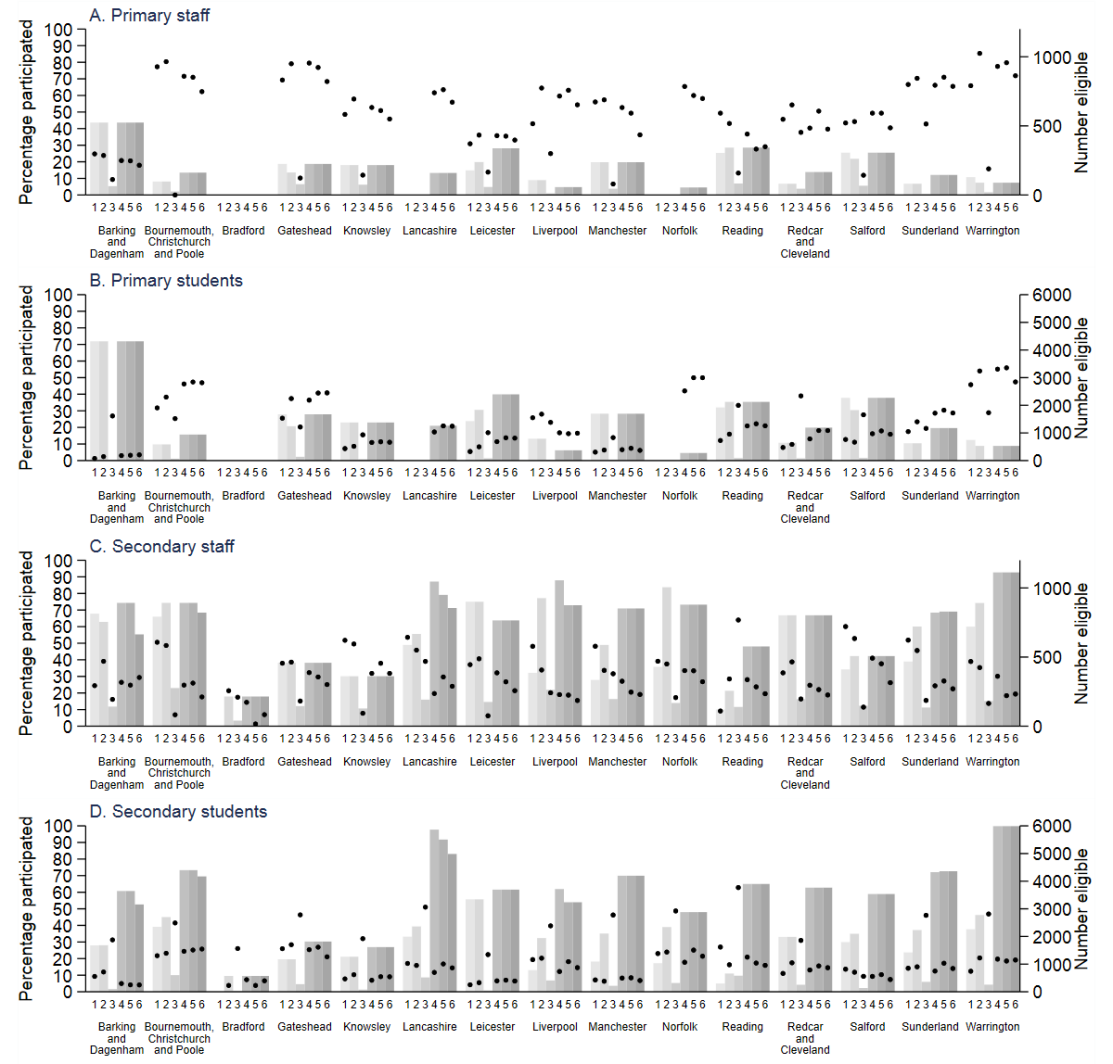
Local authority–level participation rate (percentage individuals participating of those eligible) during rounds 1 to 6 (black circles) for (A) primary school staff, (B) primary school students, (C) secondary school staff, and (D) secondary school students. Also shown are number of individuals eligible at rounds 1 to 6 (gray bars) and eligible individuals estimated from the total staff and student census in participating primary schools and the census of all staff and students in the 2 selected year groups (rounds 1 to 3) and expanded year groups (rounds 4 to 6) in participating secondary schools. Participating individuals are those who were present on the day the research team visited the school and who had at least one sample taken.

School enrollment began on October 12, 2020, with 51 primary and 74 secondary schools initially enrolled (Figures 3-6). The 2 all-through schools selected, opted to participate as both primary and secondary schools and so subsequently appeared in both samples. There were 3 LAs in which no primary schools had participated by the end of round 3 (Figures 7A and 7B). However, by the following school term (round 4), primary schools participated across all LAs, except for one (Figures 7A and 7B). Secondary schools from all 15 LAs were enrolled and participated in SIS from round 1 (Figures 7C and 7D).

Of the schools initially enrolled, following school withdrawals, schools opting out of testing rounds and enrolled schools with no registered individuals to participate in the survey rounds, 45 primary and 62 secondary schools participated in the round 1 survey, and 43 primary and 80 secondary schools participated in the round 2 survey (Figures 3-6). A total of 57 primary schools participated in rounds 4 to 6, and 91, 89, and 86 secondary schools participated in rounds 4, 5, and 6, respectively (Figures 3-6). There was a higher proportion of enrolled schools with either no participants enrolled in the survey or who opted out during rounds 1 and 2 in the autumn term than during rounds 4 to 6, especially for primary schools (Figures 3-6).

Primary schools participating in SIS were broadly representative of the sampled schools (Table 2). However, a higher proportion of sampled and participating schools appeared to be located in urban conurbations and were larger than schools across the sampled LAs. For instance, 49.2% (58/118) sampled and 50.8% (30/59) participating schools were in urban conurbations compared with 37.3% (694/1862) of schools in the sampled LAs. In addition, fewer sampled and participating schools were in the <10% students eligible for the FSMs band and the low absenteeism categories, relative to schools across sampled LAs. Again, secondary schools participating in SIS were broadly representative of the sampled schools (Table 3). Although sampled and participating schools appeared less likely to be in rural hamlets, villages, and towns than schools across sampled LAs, for other characteristics, secondary schools were more similar to those in sampled LAs.

**Table 2:** Characteristics of primary schools sampled and participating in the Schools Infection Survey (SIS) and the participants eligible and participating in SIS (any survey round).

			Schools in sampled LAs^a^ (n=1862), n (%)	Sampled schools (n=118), n (%)	Participating schools (n=59), n (%)	Eligible staff in participating schools^b^ (n=3112), n (%)	Participating staff (n=1891), n (%)	Eligible students in participating schools^b^ (n=22,225), n (%)	Participating students (n=4654), n (%)
**Location**									
	**Transmission at time of sampling**								
		Low^c^ (lower 80% LAs)	545 (29.3)	40 (33.9)	19 (32.2)	1250 (40.2)	647 (34.2)	8844 (39.8)	1523 (32.7)
		High^d^ (top 20% LAs)	1317 (70.7)	78 (66.1)	40 (67.8)	1862 (59.8)	1244 (65.8)	13,381 (60.2)	3131 (67.3)
	**Urban or rural**								
		Rural hamlet or village or town and fringe	436 (23.4)	15 (12.7)	9 (15.3)	260 (8.4)	177 (9.4)	1621 (7.3)	594 (12.8)
		Urban city and town	732 (39.3)	45 (38.1)	20 (33.9)	1176 (37.8)	739 (39.1)	7830 (35.2)	2001 (43)
		Urban conurbation	694 (37.3)	58 (49.2)	30 (50.8)	1676 (53.9)	975 (51.6)	12,774 (57.5)	2059 (44.2)
**Sociodemographics**									
	**School size (tertiles)**								
		Small: <202 students	547 (29.4)	17 (14.4)	10 (17)	230 (7.4)	179 (9.5)	1393 (6.3)	524 (11.3)
		Medium: 202 to <338 students	668 (35.9)	43 (36.4)	22 (37.3)	848 (27.2)	630 (33.3)	5258 (23.7)	1320 (28.4)
		Large: 338 to 1732 students	630 (33.8)	58 (49.2)	27 (45.8)	2034 (65.4)	1082 (57.2)	15,574 (70.1)	2810 (60.4)
		Data not available	17 (0.9)	0 (0)	0 (0)	0 (0)	0 (0)	0 (0)	0 (0)
	**Index of multiple deprivation of school postcode (2019)**								
		Most deprived	630 (33.8)	43 (36.4)	20 (33.9)	960 (30.8)	666 (35.2)	6685 (30.1)	1111 (23.9)
		2	390 (21)	26 (22)	13 (22)	846 (27.2)	378 (20)	6384 (28.7)	927 (19.9)
		3	330 (17.7)	19 (16.1)	8 (13.6)	437 (14)	282 (14.9)	3005 (13.5)	512 (11)
		4	313 (16.8)	21 (17.8)	14 (23.7)	690 (22.2)	445 (23.5)	4889 (22)	1658 (35.6)
		Least deprived	199 (10.7)	9 (7.6)	4 (6.8)	179 (5.8)	120 (6.3)	1262 (5.7)	446 (9.6)
	**Proportion of students eligible for free school meals band**								
		<10%	474 (25.5)	20 (17)	10 (17)	487 (15.6)	331 (17.5)	3467 (15.6)	1223 (26.3)
		10% to <20%	521 (28)	38 (32.2)	22 (37.3)	1078 (34.6)	689 (36.4)	7566 (34)	1769 (38)
		20% to <30%	368 (19.8)	28 (23.7)	11 (18.6)	868 (27.9)	391 (20.7)	6933 (31.2)	891 (19.1)
		30% to <40%	267 (14.3)	20 (17)	11 (18.6)	446 (14.3)	309 (16.3)	2746 (12.4)	567 (12.2)
		40% to 100%	214 (11.5)	12 (10.2)	5 (8.5)	233 (7.5)	171 (9)	1513 (6.8)	204 (4.4)
		Data not available	18 (1)	0 (0)	0 (0)	0 (0)	0 (0)	0 (0)	0 (0)
**School characteristics**									
	**Previous year school absence data (% morning or afternoon absences across year)**								
		Low (1% to 3.7%)	697 (37.4)	31 (26.3)	16 (27.1)	779 (25)	556 (29.4)	5209 (23.4)	1959 (42.1)
		Medium (3.8% to 4.6%)	641 (34.4)	44 (37.3)	22 (37.3)	1000 (32.1)	663 (35.1)	7172 (32.3)	1315 (28.3)
		High (4.7% to 48.1%)	463 (24.9)	40 (33.9)	C (C)^e^	1155 (37.1)	581 (30.7)	8418 (37.9)	1172 (25.2)
		Data not available	61 (3.3)	3 (2.5)	C (C)^e^	178 (5.7)	91 (4.8)	1426 (6.4)	208 (4.5)
	**Office for Standards in Education, Children's Services and Skills** **(OFSTED) rating**								
		Inadequate or requires improvement	150 (8.1)	13 (11)	4 (6.8)	273 (8.8)	140 (7.4)	1996 (9)	407 (8.7)
		Good	1231 (66.1)	77 (65.3)	39 (66.1)	2095 (67.3)	1221 (64.6)	15,052 (67.7)	2809 (60.4)
		Outstanding	242 (13)	18 (15.2)	10 (17)	366 (11.8)	316 (16.7)	2608 (11.7)	1064 (22.9)
		Data not available	239 (12.8)	10 (8.5)	6 (10.2)	378 (12.1)	214 (11.3)	2569 (11.6)	374 (8)
	**Student to teacher ratio**								
		<20	672 (36.1)	45 (38.1)	22 (37.3)	1182 (38)	627 (33.2)	8569 (38.6)	1442 (31)
		20 to <51	1113 (59.8)	68 (57.6)	34 (57.6)	1681 (54)	1143 (60.4)	11,890 (53.5)	2964 (63.7)
		Data not available	75 (4)	5 (4.2)	3 (5.1)	249 (8)	121 (6.4)	1766 (7.9)	248 (5.3)
	**School average progress scores in reading, writing, and mathematics^f^**								
		Low (−11.77 to −0.70)	497 (26.7)	26 (22)	12 (20.3)	481 (15.5)	318 (16.8)	3388 (15.2)	787 (16.9)
		Medium (−0.67 to 0.93)	554 (29.8)	37 (31.4)	21 (35.6)	954 (30.7)	700 (37)	6659 (30)	2108 (45.3)
		High (0.97 to 15.33)	572 (30.7)	40 (33.9)	18 (30.5)	941 (30.2)	590 (31.2)	6448 (29)	1157 (24.9)
		Data not available	239 (12.8)	15 (12.7)	8 (13.6)	736 (23.7)	283 (15)	5730 (25.8)	602 (12.9)

^a^LA: local authority.

^b^Eligible: all staff and students in schools, which participated in any of the survey rounds 1, 2, 3, 4, 5, or 6.

^c^High transmission: top 20% LAs when ranked according to SARS-CoV-2 infection per 100,000 population from September 2, 2020, to September 8, 2020.

^d^Low transmission: lower 80% LAs when ranked according to SARS-CoV-2 infection per 100,000 population from September 2, 2020, to September 8, 2020.

^e^*C*: data suppressed (2 smallest categories suppressed).

^f^Average of the combined reading, writing, and mathematics scores.

**Table 3:** Characteristics of secondary schools sampled and participating in the Schools Infection Survey (SIS) and the participants eligible and participating in SIS (any survey round).

				Schools in sampled LAs^a^ (n=368), n (%)		Sampled schools (n=185), n (%)		Participating schools (n=97), n (%)		Eligible staff in participating schools^b^ (n=12,146), n (%)		Participating staff (n=5852), n (%)		Eligible students in participating schools^c^ (n=56,519), n (%)		Participating students (n=10,188), n (%)
**Location**																
	**Transmission at time of sampling**															
		Low^d^ (lower 80% LAs)	109 (29.6)		53 (28.6)		34 (35)		4536 (37.3)		2126 (36.3)		19,748 (34.9)		4114 (40.4)	
		High^e^ (top 20% LAs)	259 (70.4)		132 (71.3)		63 (64.9)		7610 (62.7)		3726 (63.7)		36,771 (65.1)		6074 (59.6)	
	**Urban or rural**															
		Rural hamlet or village or town and fringe	44 (12)		12 (6.5)		6 (6.2)		581 (4.8)		322 (5.5)		2516 (4.5)		545 (5.3)	
		Urban city and town	169 (45.9)		76 (41.1)		47 (48.5)		5869 (48.3)		2849 (48.7)		29,227 (51.7)		6056 (59.4)	
		Urban conurbation	155 (42.1)		97 (52.4)		44 (45.4)		5696 (46.9)		2681 (45.8)		24,776 (43.8)		3587 (35.2)	
**Sociodemographics**																
	**School size (tertiles)**															
		Small: <202 students	147 (40)		65 (35.1)		35 (36.1)		3085 (25.4)		1535 (26.2)		16,280 (28.8)		2965 (29.1)	
		Medium: 202 to <338 students	128 (34.8)		66 (35.7)		37 (38.1)		4371 (36)		2279 (38.9)		22,334 (39.5)		3873 (38)	
		Large: 338 to 1732 students	90 (24.5)		C (C)^f^		25 (25.8)		4690 (38.6)		2038 (34.8)		17,905 (31.7)		3350 (32.9)	
		Data not available	3 (0.8)		C (C)^f^		0 (0)		0 (0)		0 (0)		0 (0)		0 (0)	
	**Index of multiple deprivation of school postcode (2019)**															
		Most deprived	122 (33.2)		65 (35.1)		32 (33)		3539 (29.1)		1677 (28.7)		15,666 (27.7)		2156 (21.2)	
		2	92 (25)		53 (28.6)		28 (28.9)		3902 (32.1)		1850 (31.6)		16,612 (29.4)		2459 (24.1)	
		3	56 (15.2)		24 (13)		12 (12.4)		1487 (12.2)		652 (11.1)		6383 (11.3)		1412 (13.9)	
		4	58 (15.8)		26 (14.1)		14 (14.4)		1572 (12.9)		731 (12.5)		8805 (15.6)		1601 (15.7)	
		Least deprived	40 (10.9)		17 (9.2)		11 (11.3)		1646 (13.6)		942 (16.1)		9053 (16)		2560 (25.1)	
	**Proportion of students eligible for free school meals band**															
		<10%	61 (16.6)		24 (13)		15 (15.5)		1914 (15.8)		883 (15.1)		11,675 (20.7)		3089 (30.3)	
		10% to <20%	133 (36.1)		65 (35.1)		40 (41.2)		5350 (44)		2596 (44.4)		23,006 (40.7)		4721 (46.3)	
		20% to <30%	79 (21.5)		42 (22.7)		19 (19.6)		2581 (21.2)		1206 (20.6)		11,392 (20.2)		1204 (11.8)	
		30% to <40%	60 (16.3)		36 (19.5)		14 (14.4)		1403 (11.6)		652 (11.1)		6303 (11.2)		809 (7.9)	
		40% to 100%	32 (8.7)		C (C)^f^		9 (9.3)		898 (7.4)		515 (8.8)		4143 (7.3)		365 (3.6)	
		Data not available	3 (0.8)		C (C)^f^		0 (0)		0 (0)		0 (0)		0 (0)		0 (0)	
**School characteristics**																
	**Previous year school absence data (% morning or afternoon absences across year)**															
		Low (1% to 3.7%)	27 (7.3)		15 (8.1)		9 (9.3)		1158 (9.5)		536 (9.2)		6344 (11.2)		1549 (15.2)	
		Medium (3.8% to 4.6%)	57 (15.5)		24 (13)		12 (12.4)		1358 (11.2)		607 (10.4)		7606 (13.5)		1304 (12.8)	
		High (4.7% to 48.1%)	267 (72.6)		139 (75.1)		72 (74.2)		9141 (75.3)		4510 (77.1)		40,115 (71)		7030 (69)	
		Data not available	17 (4.6)		7 (3.8)		4 (4.1)		489 (4)		199 (3.4)		2454 (4.3)		305 (3)	
	**Office for Standards in Education, Children's Services and Skills** **(OFSTED) rating**															
		Inadequate or requires improvement	88 (23.9)		46 (24.9)		19 (19.6)		2136 (17.6)		1120 (19.1)		11,203 (19.8)		1588 (15.6)	
		Good	157 (42.7)		79 (42.7)		42 (43.3)		5716 (47.1)		2876 (49.1)		24,667 (43.6)		4726 (46.4)	
		Outstanding	45 (12.2)		25 (13.5)		16 (16.5)		1949 (16)		977 (16.7)		8949 (15.8)		2450 (24)	
		Data not available	78 (21.2)		35 (18.9)		20 (20.6)		2345 (19.3)		879 (15)		11,700 (20.7)		1424 (14)	
	**Student to teacher ratio**															
		<20	329 (89.4)		164 (88.7)		83 (85.6)		10,625 (87.5)		5193 (88.7)		49,322 (87.3)		8884 (87.2)	
		20 to <51	13 (3.5)		6 (3.2)		5 (5.2)		586 (4.8)		338 (5.8)		2067 (3.7)		586 (5.8)	
		Data not available	26 (7.1)		15 (8.1)		9 (9.3)		935 (7.7)		321 (5.5)		5130 (9.1)		718 (7)	
	**School average progress scores in reading, writing, mathematics^g^**															
		Low (−1.67 to −0.19)	132 (35.9)		68 (36.8)		35 (36.1)		3855 (31.7)		2052 (35.1)		17,111 (30.3)		2655 (26.1)	
		Medium (−0.18 to 0.19)	106 (28.8)		46 (24.9)		19 (19.6)		2723 (22.4)		1245 (21.3)		11,648 (20.6)		2707 (26.6)	
		High (0.20 to 2.16)	95 (25.8)		52 (28.1)		30 (30.9)		4213 (34.7)		2058 (35.2)		20,372 (36)		4134 (40.6)	
		Data not available	35 (9.5)		19 (10.3)		13 (13.4)		1355 (11.2)		497 (8.5)		7388 (13.1)		692 (6.8)	

^a^LA: local authority.

^b^Eligible staff: all staff in participating schools at any of the survey rounds 1, 2, 3, 4, 5, or 6.

^c^Eligible students: students from the 2 consecutive years sampled in schools participating in rounds 1 to 3 and from all years (except year 11) in schools participating in rounds 4 to 6.

^d^High transmission: top 20% LAs when ranked according to SARS-CoV-2 infection per 100,000 population from September 2, 2020, to September 8, 2020.

^e^Low transmission: lower than 80% LAs when ranked according to SARS-CoV-2 infection per 100,000 population from September 2, 2020, to September 8, 2020.

^f^*C*: data suppressed (2 smallest categories suppressed).

^g^Average of the combined reading, writing, and mathematics scores.

### Individual Participation and Characteristics

The participation rate (percentage individuals participating of those eligible) was 46.6% (1159/2485) and 41.7% (3165/7583) among primary and secondary school staff, respectively, during round 1 (Figures 3-6). By round 6, the staff participation rate had declined to 42.1% (1247/2959) and 22.1% (2290/10,374) in primary and secondary schools, respectively. The primary school student participation rate increased from 12% (2164/18,066) in round 1 to 18.3% (3932/21,450) in round 6, and for secondary school students, it remained consistent at 13.6% (3053/22,478) and 14.1% (7307/51,707) in rounds 1 and 6, respectively (Figures 3-6). Overall, the staff and student participation rates were 45.2% and 16.4% in primary schools and 30% and 15.2% in secondary schools, respectively. If round 3 was excluded, 53% of the staff enrolled and consented to participate in SIS and 34% participated on the day, and 18.3% of the students enrolled and consented and 15.2% participated in the study. During round 3, in which only individuals who had enrolled but had not provided an antibody test were eligible, 18.4% (567/3079) of the staff and 42.7% (2070/4849) of the students participated.

Figures 7A-7D illustrate the LA-level participation rate within the participating schools in each round. The LA-level primary student participation rate was the highest in Warrington (Figure 7B). The LA-level secondary student participation rates were all <40% if round 3 was excluded (Figure 7D). The primary staff participation rate was highest in Warrington, Gateshead, and Bournemouth, Christchurch and Poole (Figure 7A), and the secondary staff response rate was as high as 60% in Salford (Figure 7C).

Among the participating schools, school-level characteristics were consistent between eligible and participating staff (Tables 2 and 3). This pattern was observed in both the primary and secondary schools. However, the participation of primary students was nearly 2-fold greater in rural schools than in the eligible population, with 12.8% (594/4654) of participating students versus 7.3% (1621/22,225) of eligible students attending schools in rural locations (Table 2). The same trend was observed in small schools. Participation was observed to be higher in schools with low absenteeism in the previous year and in schools with a lower percentage eligible for FSM, with 26.3% (1223/4654) of participating students in schools in <10% eligible for the FSM band, versus 15.6% (3467/22,225) of eligible students in this band. Student participation was lower in schools located in the most deprived lower layer super output areas, based on school postcode, when compared with the eligible population, and almost 2-fold higher in the least deprived areas (446/4654, 9.6%) than may be expected based on the eligible population (1262/22,225, 5.7%; Table 2). Except for the rural school location and school size, these patterns appear similar for secondary school students’ participation (Table 3).

Of those providing answers in the questionnaires, most staff who participated in SIS were female (1683/1866, 90.2% in primary and 4244/5776, 73.5% in secondary schools; Table 4). In primary schools, 56.4% (1054/1868) of staff members were aged between 35 and 54 years, whereas in secondary schools, 53.5% (3095/5785) of staff members were in this age band. In total 73.2% (5569/7605) were teaching staff, as opposed to pastoral care and administrative or maintenance staff. Just over half of the participating staff members (4026/7624, 52.8%) lived in multiple adult households with no children. A higher proportion of primary school teachers resided in the most deprived postcodes, 23% (425/1850), in contrast to secondary school teachers, 16.2% (924/5717).

Of those providing answers in the questionnaires, the gender distribution was equal across participating students, and 63.2% (9306/14,735) were in the 10- to 14-year age group. Of the primary and secondary students, 79% (3596/4553) and 86.3% (8698/10,077), respectively, were of White ethnic background (Table 4). There was a spread of participating students across school years, but <12% (1168/10,106) of secondary school students came from years 12 and 13 combined (ages 16-18 years). More than three-quarters of the participating primary school students lived in households with multiple children. A higher proportion of participating students at primary schools resided in more deprived locations, 27.5% (1257/4565) in the most deprived quintile compared with 16% (731/4565) in the least deprived quintile.

**Table 4:** Sociodemographic characteristics of individuals participating in the Schools Infection Survey (any survey round).

				Primary schools (n=59)				Secondary schools (n=97)		
				Participating primary staff^a^ (n=1891), n (%)		Participating primary students^a^ (n=4654), n (%)		Participating secondary staff^a^ (n=5852), n (%)		Participating secondary students^a^ (n=10,188), n (%)
**Demographics**										
	**Age group (years)**									
		<5	N/A^b^		346 (7.5)		N/A		N/A	
		5-9	N/A		3269 (71.1)		N/A		N/A	
		10-14	N/A		981 (21.3)		N/A		8325 (82.1)	
		≥15	N/A		N/A		N/A		1814 (17.9)	
		<35	502 (26.9)		N/A		1879 (32.5)		N/A	
		35-44	495 (26.5)		N/A		1712 (29.6)		N/A	
		45-54	559 (29.9)		N/A		1383 (23.9)		N/A	
		≥55	312 (16.7)		N/A		811 (14)		N/A	
		Age not available^c^	23		58		67		49	
	**Gender^d^**									
		Male	183 (9.8)		2322 (50.6)		1532 (26.5)		5049 (49.9)	
		Female	1683 (90.2)		2270 (49.4)		4244 (73.5)		5076 (50.1)	
		Gender not available^c^	25		62		76		63	
	**Ethnicity**									
		Asian or Asian British	96 (5.2)		528 (11.6)		222 (3.9)		617 (6.1)	
		Black African or Caribbean Black	12 (0.6)		114 (2.5)		56 (1)		222 (2.2)	
		Mixed or Multiple ethnic groups	20 (1.1)		264 (5.8)		118 (2.1)		446 (4.4)	
		Other ethnic group	6 (0.3)		51 (1.1)		30 (0.5)		94 (0.9)	
		White	1726 (92.8)		3596 (79)		5327 (92.6)		8698 (86.3)	
		Ethnicity not available^c^	31		101		99		111	
	**Job group (staff)**									
		Senior leader	172 (9.3)		N/A		453 (7.9)		N/A	
		Middle leader	115 (6.2)		N/A		1180 (20.5)		N/A	
		Teacher	491 (26.6)		N/A		2032 (35.3)		N/A	
		Teaching assistant or special educator	598 (32.4)		N/A		528 (9.2)		N/A	
		Administration or pastoral	187 (10.1)		N/A		938 (16.3)		N/A	
		Cater or clean or maintenance	147 (8)		N/A		226 (3.9)		N/A	
		Other	133 (7.2)		N/A		405 (7)		N/A	
		Job group not available^c^	48		N/A		90		N/A	
	**Year groups (students)**									
		Reception	N/A		543 (11.8)		N/A		N/A	
		Year 1	N/A		633 (13.8)		N/A		N/A	
		Year 2	N/A		672 (14.6)		N/A		N/A	
		Year 3	N/A		686 (14.9)		N/A		N/A	
		Year 4	N/A		680 (14.8)		N/A		N/A	
		Year 5	N/A		681 (14.8)		N/A		N/A	
		Year 6	N/A		696 (15.2)		N/A		N/A	
		Year 7	N/A		N/A		N/A		2477 (24.5)	
		Year 8	N/A		N/A		N/A		2587 (25.6)	
		Year 9	N/A		N/A		N/A		2220 (22)	
		Year 10	N/A		N/A		N/A		1654 (16.4)	
		Year 12	N/A		N/A		N/A		657 (6.5)	
		Year 13	N/A		N/A		N/A		511 (5.1)	
		Year group not available^c^	N/A		63		N/A		82	
**Household characteristics**										
	**Household size**									
		1-2	593 (32.2)		243 (5.3)		2313 (40.7)		740 (7.3)	
		3-5	1155 (62.6)		3749 (81.6)		3165 (55.6)		8224 (81.1)	
		≥6	96 (5.2)		605 (13.2)		211 (3.7)		1179 (11.6)	
		Household size not available^c^	47		57		163		45	
	**Household composition**									
		Only adults	950 (50.9)		C (C)^e^		3076 (53.4)		922 (9.1)	
		One child	386 (20.7)		1005 (21.6)		1116 (19.4)		3377 (33.2)	
		Multiple children	532 (28.5)		3574 (76.9)		1564 (27.2)		5883 (57.8)	
		Household composition not available^c^	23		C (C)^e^		96		6	
	**People per bedroom**									
		>2	112 (5.9)		571 (12.3)		308 (5.3)		750 (7.4)	
		>1 to 2	1128 (59.7)		3297 (70.8)		3342 (57.1)		6754 (66.3)	
		≤1	651 (34.4)		786 (16.9)		2202 (37.6)		2684 (26.3)	
		Information not available^c^	0		0		0		0	
	**Index of multiple deprivation of household postcodes 2019**									
		Most deprived	425 (23)		1257 (27.5)		924 (16.2)		2248 (22.4)	
		2	369 (19.9)		985 (21.6)		1213 (21.2)		2081 (20.7)	
		3	315 (17)		715 (15.7)		1077 (18.8)		1653 (16.4)	
		4	383 (20.7)		877 (19.2)		1259 (22)		1924 (19.1)	
		Least deprived	358 (19.4)		731 (16)		1244 (21.8)		2148 (21.4)	
		Postcode not available^c^	41		89		135		134	

^a^Data presented for all staff and students participating (providing samples for antibody or virus tests) in any of rounds 1, 2, 3, 4, and 5 or 6.

^b^N/A: not applicable.

^c^Data not available (ie, prefer not to answer or data unavailable), ≤3% for all characteristics. These are treated as missing data and therefore not included in the calculation of percentages

^d^Participants asked, *What is your gender?* (students) or *Which of the following describes how you think of yourself?* (staff), male or female or other or prefer not to say. Data are not available (ie, prefer not to answer or other or data unavailable).

^e^*C*: data suppressed (2 smallest categories suppressed).

## Discussion

### Principal Findings and Significance

The COVID-19 SIS was the largest cohort study to monitor the prevalence and transmission of SARS-CoV-2 in schools in England, a key setting and population group for the transmission of airborne infections, recruiting 22,585 participants from 59 primary and 97 secondary schools. Designed to meet an urgent policy requirement to inform pandemic response, the study aimed to characterize the extent of current and past SARS-CoV-2 infection in students and staff during the 2020-2021 academic year, including on school campuses, and investigate transmission from and to schools.

The 45.2% and 30% participation rates for staff in primary and secondary schools, respectively, are good relative to comparable studies [48] and in the context of the pandemic, although a higher uptake was predicted because of the willingness to participate in previous school-based studies [30]. The decline in staff participation across survey rounds in secondary schools (from 3165/7583, 41.7% to 2290/10,374, 22.1% between the first and last rounds) was not expected and could have been related to the increasing availability of staff vaccinations from early 2021 and a decreased perception of risk.

The overall participation rate of students was lower at an average of 15%, but this remained stable throughout the study period. This low rate could have been due to the requirement for parents to register their children via an email link, without being engaged with the process in the school. Staff participation appears resilient in terms of various school characteristics, such as whether the school is in a more deprived location, but student participation appears more sensitive to these factors. The sampling design did not seek to be representative of such factors, although it appears that a higher proportion of primary student participants resided in the most deprived postcodes at the individual level. Overall student participation was lower than might be expected in urban schools and schools in more deprived areas, which could in part indicate the digital divide and issues with sufficiently engaging parents as well as the differential ability to adhere to isolation measures if tested positive [49]. As the information, enrollment, consent, and questionnaire mechanisms were all sent via email, this could have biased enrollment toward families with greater access to electronic devices and a higher level of digital use and literacy [50]. All participating schools reported email as the primary route for communicating with parents, so it could be argued that this was unlikely to be the main factor leading to differential participation rates. However, parents affected by the digital divide would have lower access, and it was not possible for the study team to follow up with nonresponders via telephone to assist with any technological access issues.

### Strengths and Limitations

SIS has several core strengths. These include the rapid and reactive nature of the study, with regular open access bulletins, enabling the results to feed directly into policy decisions and recommendations in real time. The longitudinal nature of the study facilitated the assessment of infection and antibody conversion over time and changes in behaviors and perceptions of the COVID-19 pandemic. In addition, consequences such as long-COVID, in children especially, will be explored [51]. Furthermore, SIS is adaptable to changing circumstances. New research aspects such as vaccine coverage in staff and perceptions of the feasibility and acceptability of new policy initiatives, such as mass testing in schools and vaccination, were subsequently incorporated via short pulse follow-up questionnaires as the policy environment evolved.

In addition to comparisons with pillar 2 testing, the linkage with the CIS provides opportunities for potential comparisons between the school-based population and the community population among parallel LAs and age groups, further contextualizing the findings [21,52]. For example, as the SIS study visits were conducted on school days, the findings are representative of students and staff in school, who would be expected not to have any symptoms. These results can then be compared with estimates of community prevalence in all school-age children as well as adults between the ages of 20 and 65 years regardless of school attendance or profession [21,52 ].

However, we also reflected on the practical challenges encountered in conducting research in schools during the pandemic. First, the initial school enrollment in SIS was slow, with schools in some LAs opting not to register for SIS. In addition, until December 31, 2020, the participation rate of individuals was lower than anticipated, particularly for students, potentially introducing nonresponse bias and limiting the representativeness of the findings. This low response rate is often inherent in surveys that require participants to respond to an email or letter in the first instance. In CIS, the equivalent community infection survey, the response rate (households registered) initially decreased from 51% when inviting 20,000 households who had previously taken part in ONS surveys to 14% when opened up via Address Base to a much larger sample of households that had not been engaged in ONS surveys [53]. The Real-time Assessment of Community Transmission Study documented response rates (tests returned of letters sent out) of 20.4% for PCR testing across 13 survey rounds and of 28.9% for antibody testing across 6 rounds [54].

As these challenges were encountered, a range of design modifications were made to the recruitment procedures, including sampling additional schools in certain LAs, transitioning from a closed to an open cohort, developing paper-based communication materials to increase the accessibility of information, and providing compensation at the school level. However, there remained no compensation at the participant level, as various other community and COVID-19 studies have implemented the use of vouchers [55], and this may have contributed to attrition in response to further questionnaires among participants. The expansion of secondary school eligibility beyond the 2 original year groups to other year groups in January 2021, with the aim of increasing student recruitment, was undertaken up by 52 secondary schools in total. Although it is recognized that this would likely not address nonresponse and representativeness, it would increase the precision of school-level estimates. The result was a consistent secondary school student participation rate of approximately 14% across rounds, as recruitment increased in rounds 4 to 6, along with the eligible population.

The second challenge, in rounds 1 and 2, especially during round 2 testing in December 2020, was that several schools opted not to participate in the survey and deferred testing until the start of the spring term in January 2021. Reasons for this included testing when schools were preparing for the end of term, concerns of school disruption at this busy time, and concerns about identifying asymptomatic positives, meaning bubbles and families would have to self-isolate, and families potentially losing income or not being able to gather over the Christmas period.

Third, during round 1, home testing was not available for enrolled individuals who were not at school on the day of the survey. However, the introduction of home testing for those absent on the day of the survey from round 2 and beyond enabled participants to be included in the antibody conversion analyses. This was especially important for round 3, which was affected by the school closures, as other school studies were [56] and was modified to an exclusive home-testing round, for those without baseline antibody results.

Finally, the study relied on parental responses to questionnaires in relation to children aged <16 years. Although they were asked to complete the questionnaire in consultation with their child (the participant), it would have been preferable to survey these students directly, either in person by the survey teams during sample collection or through web-based questionnaires conducted on school computers, in terms of both the response rates and usefulness of the data.

### Conclusions

The SIS study aimed to enhance our understanding of the transmission of SARS-CoV-2 within schools by measuring past, current, and incident infection over time, the effect on outcomes such as school attendance and mental well-being, and the implementation and perceptions of control measures in schools. The findings of the ongoing analyses of these core study aims will be presented in subsequent publications with the aim of contributing substantially to the evidence base and informing future national policy.

### Source, License, Copyrights, and Credits

This work contains statistical data from the ONS which is Crown Copyright. The use of the ONS statistical data in this study does not imply endorsement of the ONS in relation to the interpretation or analysis of the statistical data. This study uses research data sets, which may not exactly reproduce national statistics aggregates. This is an open access article under the Open Government Licence.

## Appendix

Multimedia Appendix 1 Schools Infection Survey Questionnaire Bank.

## Abbreviations

CIS: COVID-19 Infection Survey
FSM: free school meal
LA: local authority
ONS: Office for National Statistics
RT-PCR: reverse transcriptase polymerase chain reaction
SIS: Schools Infection Survey
sKIDs: COVID-19 Surveillance in School KIDs
UKHSA: UK Health Security Agency

## Data Availability

Deidentified study data are available for access by accredited researchers in the Office for National Statistics Secure Research Service for accredited research purposes under part 5, chapter 5 of the Digital Economy Act of 2017. For further information about accreditation visit the Secure Research Service website.
