# Supplementary material for: The COVID-19 Schools Infection Survey in England: Protocol and Participation Profile for a Prospective Observational Cohort Study
Source: JMIR Res Protoc. 2022 Nov 10;11(11):e34075. doi: 10.2196/34075 (PMC9651002; doi:10.2196/34075)
Supplement: Multimedia Appendix 1 [file resprot_v11i11e34075_app1.pdf]

COVID-19 Schools Infection Survey (SIS) Question Bank

Contents

Enrolment Questionnaire 2
Baseline Questionnaire 5
Baseline/Follow-up questionnaire – headteachers 23
Follow-up Questionnaire 30
Vaccine Questionnaire 39
End of Survey Questionnaire 42

Enrolment Questionnaire

- **parent/guardian**
- **student over 16 years**
- **staff**

Source	Question	Response options	Routing	Notes
SIS Enrolment (parent/guardian);<br>SIS Enrolment (over 16 student)	What is the name of the school/college you/your child attends?	[enter name of school]	Ask all	
SIS Enrolment (parent/guardian);<br>SIS Enrolment (over 16 student)	What year are you/is your child in at school?	Reception / Year 0<br>Year 1<br>Year 2<br>Year 3<br>Year 4<br>Year 5<br>Year 6<br>Year 7<br>Year 8<br>Year 9<br>Year 10<br>Year 12<br>Year 13<br>Other, specify	Ask all	For SIS Enrolment (over 16 student), the only response options are 'Year 12', 'Year 13', and 'Other, specify'.<br><br>'Year 12' and 'Year 13' are not response options for SIS Enrolment (parent/guardian).
SIS Enrolment (parent/guardian);<br>SIS Enrolment (over 16 student);<br>SIS Enrolment (staff);<br>SIS Baseline (staff);<br>SIS Extended (head teacher)	What is your/your child's gender?	Male<br>Female<br>Other<br>Prefer not to say	Ask all	
SIS Enrolment (parent/guardian);<br>SIS Enrolment (over 16 student);<br>SIS Enrolment (staff)	What is your/your child's date of birth?	[enter date]	Ask all	Date entered in DD/MM/YY format.
SIS Enrolment (parent/guardian);<br>SIS Enrolment (over 16 student);<br>SIS Enrolment (staff)	What is your/your child's ethnic group?	Asian/ Asian British<br>- Indian<br>- Pakistani<br>- Bangladeshi<br>- Chinese<br>- Any other Asian background<br>Black/African/Caribbean/Black British<br>-African<br>- Caribbean<br>- Any other Black / African / Caribbean<br>White<br>- English/Welsh/Scottish/Northern Irish/British	Ask all	

Source	Question	Response options	Routing	Notes
		- Irish<br>- Gypsy or Irish Traveller<br>- Any other White background<br>Mixed/Multiple ethnic groups<br>- White and Black Caribbean<br>- White and Black African<br>- White and Asian<br>- Any other mixed / multiple ethic background<br>Other ethnic group<br>- Arab<br>- Any other ethnic group (please specify)<br>Prefer not to say		
SIS Enrolment (parent/guardian);<br>SIS Enrolment (over 16 student);<br>SIS Enrolment (staff)	What is the postcode of where you/your child lives?	[enter postcode]	Ask all	
SIS Enrolment (parent/guardian);<br>SIS Enrolment (over 16 student);<br>SIS Enrolment (staff)	In your/your child's household, how many people are there (including you or your child) in the below age groups?<br>1. Children aged 0 to 5 years old<br>2. Children aged 6 to 15 years old<br>3. Adults aged 16 to 64 years old<br>4. Adults aged 65 years old or older<br>By household, we mean the group of people your child lives with, in a single house or dwelling. If your child spends time living across two households regularly within a week, e.g. shared parental responsibilities, please count for your child's main household and the house where your child spends most of their time	[enter number of people in each age group]	Ask all	
SIS Enrolment (staff)	How many days do you work per week in this school?	[enter number of days]	Ask all	Response option selected from drop down list from 2 to 10.
SIS Enrolment (staff);<br>SIS Extended (head teacher);<br>SIS Follow-up (head teacher)	What is your job/role at the school (please tick all that apply)?	1. Headteacher / Principal<br>2. Senior leader (e.g. deputy head teacher)<br>3. Middle leader (e.g. head of department)<br>4. Teacher<br>5. Teaching assistant<br>6. Supply/locum teacher<br>7. Pastoral care<br>8. Special Education Needs (SEN) and Inclusion<br>9. Administrative/Finance/Secretarial (e.g. Business	Ask all	There are fewer response options for SIS Extended and Follow-up (head teacher) as only those in a position to answer as head teacher or on behalf of the head teacher will get the head teacher questionnaire.

Source	Question	Response options	Routing	Notes
		Managers, Administration and Accounts, IT, Media)<br>10. Maintenance (e.g. Caretaker)<br>11. Cleaning<br>12. Catering<br>13. Other (specify)		
SIS Enrolment (staff)	What year(s) do you interact with (includes teaching)? (Please tick all that apply)	Reception / Year 0<br>Year 1<br>Year 2<br>Year 3<br>Year 4<br>Year 5<br>Year 6<br>Year 7<br>Year 8<br>Year 9<br>Year 10<br>Year 11<br>Year 12<br>Year 13<br>All Years<br>Other	Ask all	
SIS Enrolment (staff)	Is there one class you spend most time with?	Yes<br>No	Ask all	
SIS Enrolment (staff)	Do you spend more time in one or more years compared to others?	Yes<br>No	Ask all	
SIS Enrolment (staff)	Do you work at any other school apart from this one (e.g. supply teacher)?	Yes<br>No	Ask all	

Baseline Questionnaire

- **parent/guardian**
- **student over 16 years**
- **staff**

Source	Question	Response options	Routing	Notes
SIS Baseline (parent/guardian)	What is your current working status?	1. Employed and currently working (including if on annual leave or sick leave for less than 4 weeks)<br>2. Employed and currently not working (including if furloughed due to the 3. COVID-19 pandemic; sick leave for 4 weeks or longer; or maternity / paternity leave)<br>4. Self-employed and currently working (including if on annual leave or sick leave for less than 4 weeks)<br>5. Self-employed and currently not working (including if furloughed due to the COVID-19 pandemic; sick leave for 4 weeks or longer; or maternity / paternity leave)<br>6. Looking for paid work and able to start<br>7. Not working and not looking for work (including voluntary work)<br>8. Retired<br>9. In full-time education	Ask all	
SIS Baseline (parent/guardian)	What is the title of your main job or business? (e.g. primary school teacher, car mechanic, district nurse, structural engineer etc.)	[enter job title]	Ask if 'Employed or self-employed and currently working'; or 'Employed or self-employed and currently not working'	
SIS Baseline (parent/guardian)	Which of these occupations / sectors do you work in? (please select one)	Armed forces<br>Arts, entertainment or recreation<br>Civil service or Local Government<br>Financial services incl. insurance<br>Food production and agriculture (incl. farming)<br>Health care<br>Hospitality (e.g. hotel, restaurant, cafe)<br>Information technology and communication<br>Manufacturing or construction<br>Other occupation sector (please specify)<br>Personal services (e.g. hairdressers, tattooists)<br>Retail sector (incl. wholesale)<br>Social care<br>Teaching and education<br>Transport (incl. storage, logistics)	Ask if 'Employed or self-employed and currently working'; or 'Employed or self-employed and currently not working'	

Source	Question	Response options	Routing	Notes
SIS Baseline (parent/guardian)	What was your work status before the first national lockdown on the 23rd March 2020?	Employed in the same occupation as currently<br>Employed in a different occupation<br>Not working and not looking for work (including if you only did voluntary work)<br>Looking for paid work and able to start immediately<br>In full-time education	Ask all	
SIS Baseline (parent/guardian)	What was your job title?	[enter job title]	Ask if 'Employed in a different occupation'	
SIS Baseline (parent/guardian)	Is there anybody else with financial responsibility for your child, whether they live in the same household or not (e.g. other parent)?	Yes<br>No<br>Do not know<br>Prefer not to say	Ask all	
SIS Baseline (parent/guardian)	What is the current working status of that other person with financial responsibility for the child?	1. Employed and currently working (including if on annual leave or sick leave for less than 4 weeks)<br>2. Employed and currently not working (including if furloughed due to the 3. COVID-19 pandemic; sick leave for 4 weeks or longer; or maternity / paternity leave)<br>4. Self-employed and currently working (including if on annual leave or sick leave for less than 4 weeks)<br>5. Self-employed and currently not working (including if furloughed due to the COVID-19 pandemic; sick leave for 4 weeks or longer; or maternity / paternity leave)<br>6. Looking for paid work and able to start<br>7. Not working and not looking for work (including voluntary work)<br>8. Retired<br>9. In full-time education	Ask if there is someone else financially responsible	
SIS Baseline (parent/guardian)	What is the title of their main job or business? (e.g. primary school teacher, car mechanic, district nurse, structural engineer etc.)	[enter job title]	Ask if other person's working status is 'Employed or self-employed and currently working'; or 'Employed or self-employed and currently not working'	
SIS Baseline (parent/guardian)	Which of these occupations / sectors do they work in? (select one)	Armed forces<br>Arts, entertainment or recreation<br>Civil service or Local Government<br>Financial services incl. insurance<br>Food production and agriculture (incl. farming)	Ask if other person's working status is 'Employed or self-employed and currently working'; or	

Source	Question	Response options	Routing	Notes
		Health care<br>Hospitality (e.g. hotel, restaurant, cafe)<br>Information technology and communication<br>Manufacturing or construction<br>Other occupation sector (please specify)<br>Personal services (e.g. hairdressers, tattooists)<br>Retail sector (incl. wholesale)<br>Social care<br>Teaching and education<br>Transport (incl. storage, logistics)	'Employed or self-employed and currently not working'	
SIS Baseline (parent/guardian);<br>SIS Baseline (over 16 student);<br>SIS Baseline (staff)	Do you/does your child currently have any chronic illness or health condition(s)?	Yes<br>No<br>Do not know	Ask all	
SIS Baseline (parent/guardian);<br>SIS Baseline (over 16 student);<br>SIS Baseline (staff)	Which ones?	Diabetes<br>Asthma<br>COPD (Chronic Obstructive Pulmonary Disease)<br>Other chronic lung condition (specify)<br>Heart condition (specify)<br>High blood pressure<br>Cancer (specify)<br>Other (specify)	Ask if have any chronic health conditions	The following response options were only included in the SIS Baseline (staff) questionnaire: COPD (Chronic Obstructive Pulmonary Disease); Other chronic lung condition (specify); High blood pressure; Cancer (specify).
SIS Baseline (staff)	Are you pregnant?	Yes<br>No<br>Do not know<br>Prefer not to say	Ask if female	
SIS Baseline (parent/guardian);<br>SIS Baseline (over 16 student);<br>SIS Baseline (staff);<br>SIS Vaccine (parent);<br>SIS Vaccine (staff/student)	Since the start of the COVID-19 pandemic, have you/has your child received a COVID-19 vaccine? (SIS Baseline)<br>or<br>Have you/has your child received any vaccination against COVID-19? (SIS Vaccine)	Yes - 1 dose<br>Yes - 2 doses<br>No<br>Prefer not to say	Ask all	SIS Vaccine has a single 'Yes' response option and includes 'Don't know' as a response option.
SIS Baseline (parent/guardian);<br>SIS Baseline (over 16 student);<br>SIS Baseline (staff)	Have you/has your child ever tested positive for COVID-19 in the past i.e. before being tested in this school survey (this can be a swab, saliva test or blood test)?	Yes<br>No	Ask all	

Source	Question	Response options	Routing	Notes
SIS Baseline (parent/guardian);<br>SIS Baseline (over 16 student);<br>SIS Baseline (staff)	What type of test was it?<br><br>Select all that apply	Nasal / throat swab test (e.g. test for current infection)<br>Saliva test (spat into cup / tube)<br>Blood test (e.g. antibody test for past infection)<br>Do not know	Ask if have tested positive for COVID-19	
SIS Baseline (parent/guardian);<br>SIS Baseline (over 16 student);<br>SIS Baseline (staff)	What was the date of the first positive nasal / throat swab or saliva test?	[enter date]	Ask if had a 'Nasal / throat swab test'	Date entered in DD/MM/YYYY format.<br><br>Only 2020 and 2021 year options available.
SIS Baseline (parent/guardian);<br>SIS Baseline (over 16 student);<br>SIS Baseline (staff)	What was the date of the first positive blood test?	[enter date]	Ask if had a 'Blood test'	Date entered in DD/MM/YYYY format.<br><br>Only 2020 and 2021 year options available.
SIS Baseline (parent/guardian);<br>SIS Baseline (over 16 student);<br>SIS Baseline (staff)	Have you/has your child had COVID-19 symptoms at any point since March 2020?	Yes<br>No<br>Do not know<br>Prefer not to say	Ask all	
SIS Baseline (parent/guardian);<br>SIS Baseline (over 16 student);<br>SIS Baseline (staff)	When did these symptoms start? (if you cannot remember the exact date, please give an estimate)	[enter date]	Ask if had COVID-19 symptoms	Date entered in DD/MM/YYYY format.<br><br>Only 2020 and 2021 year options available.
SIS Baseline (parent/guardian);<br>SIS Baseline (over 16 student);<br>SIS Baseline (staff)	Now we would like to ask about any recent symptoms:<br><br>In the week before or after the most recent samples for this school survey were collected, did you/your child experience any of the following symptoms?	Fever / chills / high temperature / hot to touch<br>New continuous cough<br>Loss or change to their sense of smell or taste<br>Shortness of breath / difficulty breathing<br>Feeling extra tired / Fatigue<br>Sore throat<br>Blocked or runny nose, sneezing<br>Muscle ache (myalgia) / Joint pain<br>Headaches<br>Nausea / vomiting<br>Tummy / Abdominal pain<br>Diarrhoea	Ask all	This question is presented in table format where, if a symptom is selected, the date of onset and duration in day(s) must be given.

Source	Question	Response options	Routing	Notes
SIS Baseline (parent/guardian);<br>SIS Baseline (over 16 student);<br>SIS Baseline (staff)	Fever / chills / high temperature / hot to touch<br>New continuous cough<br>Loss or change to their sense of smell or taste<br>Shortness of breath / difficulty breathing<br>Feeling extra tired / Fatigue<br>Sore throat<br>Blocked or runny nose, sneezing<br>Muscle ache (myalgia) / Joint pain<br>Headaches<br>Nausea / vomiting<br>Tummy / Abdominal pain<br>Diarrhoea	[enter date][select duration in days]	Ask if responded 'Yes' to any of the symptoms	Date entered in DD/MM/YYYY format.<br><br>Duration presented as a drop down list from 1 to 30.<br><br>Response required for each symptom selected.
SIS Baseline (parent/guardian);<br>SIS Baseline (over 16 student);<br>SIS Baseline (staff)	Excluding yourself/your child, how many people live in this household?	[select number of people]<br>Prefer not to say	Ask all	Response options presented as a drop down list from 1 to 10.<br><br>Question wording slightly different for SIS Baseline (parent/guardian) as the parent/guardian answering the questionnaire is included in number of people in household.
SIS Baseline (parent/guardian);<br>SIS Baseline (over 16 student);<br>SIS Baseline (staff)	Relationship to yourself/the participating child:	Parent (Mother / Stepmother / Father / Stepfather)<br>Grandparent<br>Aunt / Uncle<br>Sibling (Brother / stepbrother / Sister / Stepsister)<br>Guardian<br>Other relative<br>No relation	Ask if selected any other people in household<br><br>Asked for each member of household	Response options presented as drop down list.
SIS Baseline (parent/guardian);<br>SIS Baseline (over 16 student);<br>SIS Baseline (staff)	What is their gender?	Male<br>Female<br>Other<br>Prefer not to say	Ask if selected any other people in household<br><br>Asked for each member of household	
SIS Baseline (parent/guardian);<br>SIS Baseline (over 16 student);<br>SIS Baseline (staff)	What is their date of birth? (If unsure, please skip this and only enter their age group below)	[enter date]	Ask if selected any other people in household<br><br>Asked for each member of household	Date entered in DD/MM/YYYY format

Source	Question	Response options	Routing	Notes
SIS Baseline (parent/guardian);<br>SIS Baseline (over 16 student);<br>SIS Baseline (staff)	What is their age group?	0-4 years<br>5-9 years<br>10-14 years<br>15-19 years<br>20-24 years<br>25-29 years<br>30-34 years<br>35-39 years<br>40-44 years<br>45-49 years<br>50-54 years<br>55-59 years<br>60-64 years<br>65-69 years<br>70-74 years<br>75-79 years<br>80-84 years<br>85-89 years<br>90 years and above<br>Prefer not to say<br>Unknown	Ask if selected any other people in household<br><br>Asked for each member of household	
SIS Baseline (parent/guardian);<br>SIS Baseline (over 16 student);<br>SIS Baseline (staff)	What is their current working status?	1. Employed and currently working (including if on annual leave or sick leave for less than 4 weeks)<br>2. Employed and currently not working (including if furloughed due to the COVID-19 pandemic; sick leave for 4 weeks or longer, or maternity / paternity leave)<br>3. Self-employed and currently working (including if on annual leave or sick leave for less than 4 weeks)<br>4. Self-employed and currently not working (including if furloughed due to the COVID-19 pandemic; sick leave for 4 weeks or longer or maternity / paternity leave)<br>5. Looking for paid work and able to start<br>6. Not working and not looking for work (including voluntary work)<br>7. Retired<br>8. Child under 5 years old not attending nursery, pre-school, childminder<br>9. Child under 5 years old attending nursery, pre-school, childminder<br>10. In full time education (including children over 4 years and adults)	Ask if selected any other people in household<br><br>Asked for each member of household	
SIS Baseline (parent/guardian);<br>SIS Baseline (over 16 student);<br>SIS Baseline (staff)	Are they attending the same school where you attend/as the participating child? (parent/guardian and over 16 student)<br><br>Are they attending the school where you work? (staff)	Yes<br>No	Ask if member is in full-time education<br><br>Asked for each member of household	

Source	Question	Response options	Routing	Notes
SIS Baseline (parent/guardian);<br>SIS Baseline (over 16 student);<br>SIS Baseline (staff)	Which of the following applies to their work situation	1. Works in a social care setting (e.g. nursing care home, residential care home, home carer, day centre)<br>2. Works in a healthcare setting (e.g. GP or Dental practice, Hospital)<br>3. Works in a retail, hospitality or transport setting (e.g. shop worker, bus driver, courier)<br>4. Works in early years education, a school or post-16 education setting (e.g. nursery worker, primary or secondary school or college teaching, assisting or cleaning, feeding)<br>5. Works in another job	Ask if member is 'Currently employed or self-employed and working', or 'Currently employed or self-employed and not working'<br><br>Asked for each member of household	
SIS Baseline (parent/guardian);<br>SIS Baseline (over 16 student);<br>SIS Baseline (staff)	Does their job / role primarily involve direct contact, in person, with patients / clients / residents / service users on a day to day basis?	Yes<br>No<br>Prefer not to say	Ask if member is 'Currently employed or self-employed and working', or 'Currently employed or self-employed and not working'<br><br>Asked for each member of household	
SIS Baseline (parent/guardian);<br>SIS Baseline (over 16 student);<br>SIS Baseline (staff)	Have they ever tested positive for COVID-19 before your/your child's most recent testing in this school survey (this can be either a swab, saliva test or a blood test)?	Yes<br>No<br>Do not know	Ask if selected any other people in household<br><br>Asked for each member of household	
SIS Baseline (parent/guardian);<br>SIS Baseline (over 16 student);<br>SIS Baseline (staff)	What type of test was it?<br><br>Select all that apply	Nasal / throat swab test (e.g. test for current infection)<br>Saliva test (spat into cup / tube)<br>Blood test (e.g. antibody test for past infection)<br>Do not know	Ask if member had a COVID-19 test<br><br>Asked for each member of household	
SIS Baseline (parent/guardian);<br>SIS Baseline (over 16 student);<br>SIS Baseline (staff)	What was the date of the first positive test?	[enter date for each test type]	Ask if member had a COVID-19 test<br><br>Asked for each member of household	Date entered in DD/MM/YYYY format.<br><br>Only 2020 and 2021 year options available.
SIS Baseline (parent/guardian);<br>SIS Baseline (over 16 student)	Does your family own a car or another motorised vehicle?	Yes<br>No	Ask all	

Source	Question	Response options	Routing	Notes
SIS Baseline (parent/guardian);<br>SIS Baseline (over 16 student)	Are you/is your child entitled to receive free school meals?	Yes<br>No<br>Do not know<br>Prefer not to say	Ask all	
SIS Baseline (parent/guardian);<br>SIS Baseline (over 16 student);<br>SIS Baseline (staff)	Have you/has your child travelled abroad since March 2020?	Yes<br>No<br>Prefer not to say	Ask all	
SIS Baseline (parent/guardian);<br>SIS Baseline (over 16 student);<br>SIS Baseline (staff)	How many times have you/they travelled abroad?	[select number of times]	Ask if have travelled abroad	Response option presented as drop down list from 1 to 10.
SIS Baseline (parent/guardian);<br>SIS Baseline (over 16 student);<br>SIS Baseline (staff)	Which country or countries did you/they visit?	[enter name of country or countries]	Ask if have travelled abroad	
SIS Baseline (parent/guardian);<br>SIS Baseline (over 16 student);<br>SIS Baseline (staff)	When did you/they leave the UK?	[enter date]	Ask if have travelled abroad	Date entered in DD/MM/YYYY format.<br><br>Only 2020 and 2021 year options available.
SIS Baseline (parent/guardian);<br>SIS Baseline (over 16 student);<br>SIS Baseline (staff)	When did you/they return to the UK?	[enter date]	Ask if have travelled abroad	Date entered in DD/MM/YYYY format.<br><br>Only 2020 and 2021 year options available.
SIS Baseline (parent/guardian);<br>SIS Baseline (over 16 student);<br>SIS Baseline (staff)	Since the start of this term, how many days per week have you/has your child usually attended your/their school? (parent/guardian and over 16 student)<br><br>Since the start of this term, how many days per week have you worked in-person at school? (staff)	[select number of days]	Ask all	Response option presented as drop down list from 1 to 7.

Source	Question	Response options	Routing	Notes
SIS Baseline (parent/guardian);<br>SIS Baseline (over 16 student);<br>SIS Baseline (staff)	How have you/has your child mainly travelled to and from school in the current term?	Walking<br>Bicycle, motorbike<br>Moped or scooter<br>Dedicated school bus<br>Public transport bus<br>Public transport train (including train, Tram, DLR, overground) Public transport underground<br>Car with only yourself or shared with household members<br>Car shared with people outside your household<br>Taxicab, Uber, etc.<br>Other (please specify)	Ask all	
SIS Baseline (staff)	How many times have per week have you used any public transport in the current term?<br>(Please count each journey (to or from school) separately; for example, if you have taken public transport to go to school 5 mornings a week but return from school on foot, that counts as 5 times per week).	[select number]	Ask if selected any public transport	Response option presented as drop down list from 0 to 25.
SIS Baseline (parent/guardian);<br>SIS Baseline (over 16 student);<br>SIS Baseline (staff)	When travelling to and from school, which of the following applied? If possible, please consult your child to answer this question.<br>1. You/your child wore a face mask or face covering during trips on public transport<br>2. You/your child observed social distancing rules during trips on public transport	1. Always<br>2. Sometimes<br>3. Rarely<br>4. Never<br>5. Prefer not to say	Ask if selected any public transport	Response option required for use of face covering and social distancing.
SIS Baseline (parent/guardian);<br>SIS Baseline (over 16 student)	Do you/does your child sit in their bubble when traveling on the dedicated school bus? If possible, please consult your child to answer this question.	Yes<br>No<br>Sometimes<br>Do not know	Ask if child uses dedicated school bus	

Source	Question	Response options	Routing	Notes
SIS Baseline (parent/guardian);<br>SIS Baseline (over 16 student);<br>SIS Baseline (staff)	While at school, which of the following apply?<br><br>1. You/your child wore a face mask or face covering out of class when mixing predominantly with students e.g. corridors, playground, sports / music / art activities<br><br>2. You have worn a face mask or face covering out of class when mixing predominantly with other staff e.g. in the staff room / dining room<br><br>3. You/your child used hand sanitiser while in school, between classes<br><br>4. You/your child used hand sanitiser while in school, during classes<br><br>5. You/your child washed their hands with soap regularly (three or more times a day)<br><br>6. You/your child followed social distancing rules with pupils/while in school<br><br>7. You have followed social distancing rules with other staff members	1. Always<br>2. Sometimes<br>3. Rarely<br>4. Never<br>5. Prefer not to say	Ask all	Response option required for all protective measures.<br><br>Response options 2 and 7 only applies to SIS Baseline (staff) and response option 1 wording only includes "…when mixing predominantly with students…" for SIS Baseline (staff).

Source	Question	Response options	Routing	Notes
SIS Baseline (parent/guardian); SIS Baseline (over 16 student)	On a typical day at school, how many different people from your/their school (excluding any members of your household) would you/your child talk to face-to-face either one-to-one or in a small group (i.e. not speaking to the whole class) in each of the following places?<br><br>If you/your child would normally talk to somebody in more than one place (for example they might talk to their friend at school and in a neighbourhood club) please only count them for the place where they spend the most time with them.<br><br>1. People who are in your/their class<br><br>2. People who are in your/their year group, but a different class<br><br>3. People who are in a year group different from yours/theirs<br><br>4. People who are in a year group next to yours/theirs (below or above)<br><br>5. Adults (for example teachers, classroom assistants, other staff)	None<br>1 to 5<br>6 to 10<br>11 or more<br>Do not know<br>Prefer not to say	Ask all	Response options provided across three different settings: At school; At after school clubs; Anywhere else<br><br>Response required for all groups of people the child interacts with
SIS Baseline (staff)	On a typical day when you are at school, how many different people from your school would you talk to face-to-face either one-to-one or in a small group in each of the following places?<br><br>1. Pupils from your school<br><br>2. Staff members from your school<br><br>If you would normally talk to somebody in more than one place (for example you might talk to your colleague out of school and at school) please only count them once, in the place where you spent the most time with them.	None<br>1 to 5<br>6 to 10<br>11 or more<br>Do not know<br>Prefer not to say	Ask all	Response options provided across three different settings: At school; After school activities; Anywhere else.<br><br>Response required for all groups of people the child interacts with.

Source	Question	Response options	Routing	Notes
SIS Baseline (parent/guardian); SIS Baseline (over 16 student)	Thinking of up to six people you/your child spends the most time with at school, how many of the (up to) six are in:<br><br>1. your/their year group and same class<br><br>2. your/their year group and different class<br><br>3. the year group directly above yours/theirs<br><br>4. the year group directly below yours/theirs<br><br>5. other year groups?	[select number of people]<br>Do not know	Ask all	Response option presented as drop down list from 1 to 6 with 'Do not know' as the last option.<br><br>Response required for each social group.
SIS Baseline (parent/guardian); SIS Baseline (over 16 student)	In the past four weeks, how many times a week, typically, have you/has your child taken part in the following indoor extra-curricular activities, which have involved in-person interaction or contact, with people outside your household?<br><br>1. Youth clubs, scouts, girl guides, music, art or other organised activities<br><br>2. Voluntary or community work<br><br>3. Sport, dance<br><br>4. Tutorials (e.g. for school subjects) or religious classes<br><br>5. Other	Never<br>Less than 1 per week<br>Once per week<br>2 times per week<br>3 times per week<br>4 or more times per week<br>Do not know	Ask all	Response required for each extra-curricular activity.
SIS Baseline (parent/guardian); SIS Baseline (over 16 student)	Thinking of the past four weeks, apart from the usual extra-curricular activities asked about just now, did you/your child have indoor contact with anyone from outside your household on a regular (at least once a week) basis?	Yes<br>No<br>Prefer not to say	Ask all	
SIS Baseline (parent/guardian); SIS Baseline (over 16 student)	Is that:	A friend<br>Grandparents<br>A cleaner<br>Childminder<br>Other (please specify)	Ask if had indoor contact	Childminder' is not a response option for SIS Baseline (over 16 student).

Source	Question	Response options	Routing	Notes
SIS Baseline (parent/guardian);<br>SIS Baseline (over 16 student);<br>SIS Baseline (staff)	Thinking of the past 4 weeks, have you/has your child or anyone who usually lives in your/their household(s) been inside a hospital, nursing care home or residential care home for any reason (e.g. for work, treatment, to visit someone?	Yes, I/they have<br>Yes, someone from my/their household has<br>No, nobody has<br>Not sure	Ask all	
SIS Baseline (parent/guardian);<br>SIS Baseline (over 16 student);<br>SIS Baseline (staff)	Which of the following places did you/your child visit in the past 4 weeks?<br><br>1. Different household (e.g. visiting a friend's or relative's house)<br>2. Grocery store / shops / supermarkets<br>3. Cinema / Theatre / Concert / Music venue<br>4. Restaurant / Café / Pub / food outlet – indoor<br>5. Place of worship (church, mosque, temple, synagogue, etc.)<br>6. Indoor leisure centre / sports centre / gym / swimming pool<br>7. Outdoor recreation area (e.g. Parks / playing fields / water sports / outdoor pool) with people outside your household<br>8. Hairdresser / Beauty Salon / Tanning studio<br>9. Used public transport (bus, tube, train, plane etc.)<br>10. Attending a party (including weddings and christenings, etc.)<br>11. Other public place (museum, art gallery etc.)	Every day or most days<br>More than once a week but not every day<br>About once a week<br>Once a month or more, but less than once a week<br>Never<br>Do not know<br>Prefer not to say	Ask all	Response required for each place.

SIS Baseline (parent/guardian);<br>SIS Baseline (over 16 student)	Which of these phrases best describes you/your child?<br><br>For each item, please mark the box for 'Not True', 'Somewhat True' or 'Certainly True'. It would help us if you answered all items as best you can even if you are not absolutely certain or the item seems daft! Please give your answers on the basis of how things have been for you/your child's behaviour over the last six months<br>1. Considerate of other people's feelings<br>2. Restless, overactive, cannot stay still for long<br>3. Often complains of headaches, stomach-aches or sickness<br>4. Shares readily with other children (treats, toys, pencils etc.)<br>5. Often has temper tantrums or hot tempers<br>6. Rather solitary, tends to play alone<br>7. Generally obedient, usually does what adults request<br>8. Many worries, often seems worried<br>9. Helpful if someone is hurt, upset or feeling ill<br>10. Constantly fidgeting or squirming<br>11. Has at least one good friend<br>12. Often fights with other children or bullies them<br>13. Often unhappy, down-hearted or tearful<br>14. Generally liked by other children<br>15. Easily distracted, concentration wanders<br>16. Nervous or clingy in new situations, easily loses confidence<br>17. Kind to younger children<br>18. Often lies or cheats<br>19. Picked on or bullied by other children<br>20. Often volunteers to help others (parents, teachers, other children)<br>21. Thinks things out before acting<br>22. Steals from home, school or elsewhere<br>23. Gets on better with adults than with other children<br>24. Many fears, easily scared<br>25. Sees tasks through to the end, good attention span	Not True<br>Somewhat True<br>Certainly True	Ask all	Response required for each of the 25 phrases.<br><br>Question wording slightly different for SIS Baseline (over 16 student).<br><br>Includes the following intro: "We want to understand the impact of school closures and re-openings on young people's mental wellbeing and behaviour."

SIS Baseline (staff);<br>SIS Follow-up (staff)	We want to understand the impact of school closures and re-opening on staff wellbeing. Below are some statements of job-related feelings. Please read each statement carefully and decide if you ever feel this way about your job.<br>1. I feel emotionally drained by my work<br>2. I feel used up at the end of the workday<br>3. I feel fatigued when I get up in the morning and have to face another day on the job<br>4. I can easily understand how my students feel about things<br>5. I feel I treat some students as if they were impersonal objects.<br>6. Working with people all day is really a strain for me<br>7. I deal very effectively with the problems of my students<br>8. I feel burned out from my work<br>9. I feel I'm positively influencing other people's lives through my work<br>10. I've become more callous toward people since I took this job<br>11. I worry that this job is hardening me emotionally<br>12. I feel very energetic<br>13. I feel frustrated by my job<br>14. I feel I'm working too hard in my job<br>15. I don't really care what happens to some students<br>16. Working directly with people puts too much stress on me<br>17. I can easily create a relaxed atmosphere with my students<br>18. I feel exhilarated after working closely with my students<br>19. I have accomplished many worthwhile things in this job<br>20. I feel like I'm at the end of my tether<br>21. In my work, I deal with emotional problems very calmly<br>22. I feel students blame me for some of their problems	0 – Never<br>1 – A few times a year or less<br>2 – Once a month or less<br>3 – A few times a month<br>4 – Once a week<br>5 – A few times a week<br>6 – Everyday	Ask all	Response option not mandatory.<br><br>Question wording slightly different for SIS Follow-up (staff), including further elaboration on the response options.

Source	Question	Response options	Routing	Notes
SIS Baseline (over 16 student);<br>SIS baseline (staff)	Do you currently have a job (e.g. part-time, evening or week-end job) apart from your studies? (over 16 student)<br><br>Currently, do you have any other employment, or a second job, apart from your role at the school? (staff)	1. No, I do not have a job<br>2. Yes, I also work in a social care setting (e.g. nursing care home, residential care home, home carer, day centre)<br>3. Yes, I also work in a healthcare setting (e.g. GP or Dental practice, Hospital)<br>4. Yes, I also work in a retail, hospitality or transport setting (e.g. shop worker, bus driver, courier)<br>5. Yes, I also work in early years education, a school or post-16 education setting (e.g. nursery worker, primary or secondary school or college teaching, assisting or cleaning, feeding)<br>6. Yes, I also work in another job	Ask all	
SIS Baseline (over 16 student);<br>SIS baseline (staff)	Does your role primarily involve direct contact, in person, with patients / clients / residents / service users on a day-to-day basis?	Yes<br>No	Ask if work in social care or healthcare setting	
SIS Baseline (over 16 student);<br>SIS baseline (staff)	Does that role primarily involve direct contact, in person, with clients / customers on a day-to-day basis?	Yes<br>No	Ask if work in retail, hospitality or transport setting	
SIS Baseline (over 16 student);<br>SIS baseline (staff)	Does that role primarily involve direct contact, in person, with infants, young children or students on a day-to-day basis?	Yes<br>No	Ask if work in education setting	
SIS Baseline (staff)	Does this role primarily involve direct contact, in person, with customers / guests / service-users on a day-to-day basis?	Yes<br>No	Ask if work in another type of job	
SIS Baseline (over 16 student)	Do you mostly work…	from home<br>outside of the home<br>both at home and outside of the home	Ask if work in another type of job	
SIS Baseline (over 16 student)	Since the start of this term, have you been assigned to a group or 'bubble' that generally does not mix with other groups?	Yes, in a bubble smaller than normal class size<br>Yes, in a bubble that is normal class size<br>Yes, in a bubble of the entire year group<br>No<br>I do not know<br>Other, please specify	Ask all	

Source	Question	Response options	Routing	Notes
SIS Baseline (over 16 student)	Are there any times in the day when students in your bubble might mix with other bubbles or groups?	Yes, during break<br>Yes, during lunch<br>Yes, during sports<br>Yes, hanging out immediately after school<br>No<br>Do not know<br>Prefer not to say	Ask if 'Yes' or 'Other' in response to bubble	
SIS Baseline (over 16 student)	In your experience, how well are the following preventive measures being followed by other students?<br>1. Students wear facemasks or face coverings in corridors or communal areas<br>2. Students maintain distance from other students<br>3. Students regularly (three or more times a day) wash or sanitise hands<br>4. Students catch coughs or sneezes with tissue or arm	Always<br>Sometimes<br>Rarely<br>Never<br>Do not know	Ask all	Response required for each preventive measure.
SIS Baseline (staff)	In your experience, how well are the preventive measures being followed? Please answer questions about school-related activities and behaviours thinking of the current term<br>1. Staff wear facemasks or face coverings in the classroom<br>2. Staff wear facemasks or face coverings in corridors or communal areas<br>3. Staff wear facemasks or face coverings in staff rooms or shared office space<br>4. Staff maintain 2m distance from students<br>5. Staff maintain 2m distance from other adults at school<br>6. Staff regularly wash or sanitise hands	Always<br>Sometimes<br>Rarely<br>Never<br>Not applicable<br>Do not know	Ask all	

Source	Question	Response options	Routing	Notes
SIS Baseline (staff)	In your experience, how well are the preventive measures being followed? Please answer questions about school-related activities and behaviours thinking of the current term<br>1. Students wear facemasks or face coverings in the classroom<br>2. Students regularly wash or sanitise hands<br>3. Students catch coughs or sneezes with tissue or arm	Always<br>Sometimes<br>Rarely<br>Never<br>Not applicable<br>Do not know	Ask all	
SIS Baseline (staff)	In your experience, how well are the preventive measures being followed? Please answer questions about school-related activities and behaviours thinking of the current term<br>1. Seats and desks are spaced apart to maintain distance between students<br>2. All desks face forward<br>3. Increased cleaning of frequently touched surfaces<br>4. Students do not share equipment or learning materials in classrooms<br>5. Students do not carry equipment or learning materials between home and school	Always<br>Sometimes<br>Rarely<br>Never<br>Not applicable<br>Do not know	Ask all	
SIS Baseline (staff)	Is your role at the school a Teacher or a Teaching Assistant?	Yes<br>No	Ask teachers and teaching assistants only	This question should only be presented to those who selected 'Teacher' or 'Teaching assistant' in the SIS Enrolment (staff) questionnaire.
SIS Baseline (staff)	Are there times in the day when student might mix with students in other bubbles or groups?<br><br>Please answer questions about school-related activities and behaviours thinking of the current term.	Yes, during break<br>Yes, during lunch<br>Yes, during sports<br>Yes, other (please specify)<br>No<br>Do not know<br>Prefer not to say<br>Not applicable	Ask teachers and teaching assistants only	

Baseline/Follow-up questionnaire – headteachers

Source	Question	Response options	Routing	Notes
SIS Baseline (head teacher)	What is the full name of the school?	[enter name of school]	Ask all	
SIS Baseline (head teacher)	Which year groups does your school include?	Nursery<br>Reception<br>Years 1-2<br>Years 3-6<br>Years 7-9<br>Years 10-11<br>Years 12-13	Ask all	Multiple response options allowed.
SIS Enrolment (parent/guardian);	What is your/your child's gender?	Male<br>Female<br>Other<br>Prefer not to say	Ask all	
SIS Enrolment (over 16 student);	What is your age?	25 years and under<br>26-35 years<br>36-45 years<br>46-55 years<br>56 years or over<br>Prefer not to say	Ask all	
SIS Enrolment (staff);	What is your job/role at the school (please tick all that apply)?	1. Headteacher / Principal<br>2. Senior leader (e.g. deputy head teacher)<br>3. Middle leader (e.g. head of department)<br>4. Teacher<br>5. Teaching assistant<br>6. Supply/locum teacher<br>7. Pastoral care<br>8. Special Education Needs (SEN) and Inclusion<br>9. Administrative/Finance/Secretarial (e.g. Business Managers, Administration and Accounts, IT, Media)<br>10. Maintenance (e.g. Caretaker)<br>11. Cleaning<br>12. Catering<br>13. Other (specify)	Ask all	There are fewer response options for SIS Extended and Follow-up (head teacher) as only those in a position to answer as head teacher or on behalf of the head teacher will get the head teacher questionnaire.
SIS Baseline (staff);	Did your school remain open for children of key workers and/or vulnerable children during the national lockdown period (23 March – 31 May 2020)?	Yes<br>No<br>Prefer not to say	Ask all	
SIS Baseline (head teacher)	Was your school open for any students after the lockdown from early June until the end of the summer term 2020?	Yes<br>No<br>Prefer not to say	Ask all	

Source	Question	Response options	Routing	Notes
SIS Baseline (head teacher)	Does your school keep students in consistent student groups or ‘bubbles’ that do not mix with other student groups?	Yes, in bubbles smaller than normal class sizes<br>Yes, in bubbles that are normal class sizes<br>Yes, in bubbles of an entire year group<br>No<br>Other, please specify	Ask all	
SIS Enrolment (staff);	How far apart from each other are students generally seated in the classroom?	Less than 0.5 metre apart<br>Over 0.5m but under 1m apart<br>Over 1m but under 2m apart<br>Over 2m apart<br>Other, please specify<br>Do not know<br>Prefer not to say	Ask all	
SIS Baseline (head teacher);	Which of the following staff preventive measures are currently being implemented at your school? Tick all that apply<br>In your opinion, how challenging is this measure to implement at your school?<br>1. Staff stay home if they, or someone in their household, has COVID-19 symptoms<br>2. Staff do not attend work (may work from home) if clinically vulnerable<br>3. Staff do not attend work (may work from home) if they live with someone who is clinically vulnerable<br>4. Staff work from home if their job can be effectively done from home<br>5. Staff wear facemasks or face coverings in the classroom<br>6. Staff wear facemasks or face coverings in corridors or communal areas<br>7. Staff wear facemasks or face coverings in staff rooms or shared office space<br>8. Stop in-person staff meetings<br>9. Staff maintain 2m distance from students<br>10. Staff maintain 2m distance from other adults at school<br>11. Teachers stay at the front of the class<br>12. Staff regularly wash (three or more times a day) or sanitise hands	Has been implemented<br>Has not been implemented<br>Not applicable<br>Not sure<br><br>and<br><br>Major challenges to implement<br>Some challenges to implement<br>Easy to implement	Ask all	This question is presented in table format where, if a measure is being implemented, the respondent needs to specify how challenging the measure is to implement.<br><br>Due to the number of measures listed, they have been separated according to the following categories: Staff, Students, Classroom environment, School rules and environment.

Source	Question	Response options	Routing	Notes
SIS Follow-up (head teacher)	Which of the following student preventive measures are currently being implemented at your school? Tick all that apply<br><br>In your opinion, how challenging is this measure to implement at your school?<br><br>1. Students stay home if they, or someone in their household, has COVID-19 symptoms<br><br>2. Students wear face masks or face coverings in the classroom<br><br>3. Students wear facemasks or face coverings in corridors or communal areas<br><br>4. Students wear facemasks on dedicated school transport<br><br>5. Students stay in the same group ('bubble') at all times during the school day<br><br>6. Students stay in the same group (‘bubble’) in wraparound care as in the classroom<br><br>7. Students stay in the same group or (‘bubble’) on school transport as in the classroom<br><br>8. Not providing wraparound care<br><br>9. Not providing after-school activities<br><br>10. The same teachers work with a single class throughout the school week<br><br>11. The same teaching assistants work with a single class throughout the school week<br><br>12. Temperature checks for students at least once a week<br><br>13. Students maintain distance from other students within their group (‘bubble’)<br><br>14. Students maintain distance from other students outside of their group (‘bubble’)<br><br>15. Students regularly wash (three or more times a day) or sanitise hands<br><br>16. Students catch coughs or sneezes with tissue or arm	Has been implemented<br>Has not been implemented<br>Not applicable<br>Not sure<br><br>and<br><br>Major challenges to implement<br>Some challenges to implement<br>Easy to implement	Ask all	

Source	Question	Response options	Routing	Notes
SIS Baseline (head teacher)	Which of the following classroom environment preventive measures are currently being implemented at your school? Tick all that apply<br>In your opinion, how challenging is this measure to implement at your school?<br>1. Additional hand sanitisers in classrooms<br>2. Seats and desks are spaced apart to maintain distance between students<br>3. Keep all desks facing forward<br>4. Remove non-essential objects from classrooms<br>5. Removing soft furnishings and toys that are hard to clean<br>6. Increased cleaning of frequently touched surfaces<br>7. Scheduling more outdoor lessons and activities<br>9. Ensuring students use the same classroom throughout the day<br>10. Ensuring students do not share equipment or learning materials in classrooms<br>11. Discouraging students from carrying equipment or learning materials between home and school other than where essential<br>12. Windows are open all of the time<br>13. Windows are opened periodically to air the classroom<br>14. Mechanical ventilation systems (if present) are run continuously	Has been implemented<br>Has not been implemented<br>Not applicable<br>Not sure<br><br>and<br><br>Major challenges to implement<br>Some challenges to implement<br>Easy to implement	Ask all	

Source	Question	Response options	Routing	Notes
SIS Baseline (head teacher)	Which of the following school rule and environment preventive measures are currently being implemented at your school? Tick all that apply<br><br>In your opinion, how challenging is this measure to implement at your school?<br><br>1. One-way systems in school corridors<br><br>2. Hand sanitisers at the school entrance<br><br>3. Staggering the times students start and end the school day<br><br>4. Staggering break times for different classes<br><br>5. Clean surfaces in the dining hall between groups<br><br>6. Stop large gatherings of students e.g. assemblies<br><br>7. Stop team sports<br><br>8. Distancing among parents dropping off or picking up children<br><br>9. Toilet facilities are restricted to particular bubbles	Has been implemented<br>Has not been implemented<br>Not applicable<br>Not sure<br><br>and<br><br>Major challenges to implement<br>Some challenges to implement<br>Easy to implement	Ask all	
SIS Baseline (head teacher);	Which preventive measures have been particularly challenging to implement, or have not worked well?	[free text answer]	Ask all	
SIS Follow-up (head teacher)	Have you conducted or do you plan to conduct an internal evaluation or review of how well preventive measures are being implemented at your school?	Yes<br>No<br>Do not know<br>Prefer not to say	Ask all	
SIS Baseline (head teacher)	Approximately how often are these internal evaluations/reviews being, or going to be, conducted?	About once or twice per week<br>About once or twice per month<br>About once or twice per term or less<br>Other, please specify<br>Do not know<br>Prefer not to say	Ask if plan on conducting review	
SIS Baseline (head teacher);	Have you received information on how to reduce risk of transmission of COVID-19 in schools from any of the following sources?	Department for Education<br>Public Health England<br>Local authority<br>Other, please specify<br>No information received<br>Do not know<br>Prefer not to say	Ask all	Multiple response options allowed.

Source	Question	Response options	Routing	Notes
SIS Follow-up (head teacher)	How would you rate the usefulness of this information?	Very useful<br>Quite useful<br>Not very useful<br>Not at all useful<br>Do not know / Prefer not to say	Ask if received information	
SIS Baseline (head teacher);	Does your school have a process in place if a staff member or student reports COVID-19 symptoms while at school?	Yes<br>No<br>Do not know<br>Prefer not to say	Ask all	
SIS Follow-up (head teacher)	Does your school have a process in place if a staff member or student reports that they, or someone in their household, has COVID-19 symptoms?	Yes<br>No<br>Do not know<br>Prefer not to say	Ask all	
SIS Baseline (head teacher);	Does your school have a policy about the number of cases that would necessitate closure of a class?	Yes, based on number of cases; please enter number<br>Yes, based on fixed number of cases; please specify<br>No<br>Do not know<br>Prefer not to say	Ask all	Response options are slightly different for SIS Extended (head teacher) as there is only a single 'Yes' response.
SIS Follow-up (head teacher)	Does your school have a policy about the number of cases that would necessitate closure of a year group, or other bubble?	Yes, based on number of cases; please enter number<br>Yes, based on fixed number of cases; please specify<br>No<br>Do not know<br>Prefer not to say	Ask all	Response options are slightly different for SIS Extended (head teacher) as there is only a single 'Yes' response.
SIS Baseline (head teacher);	Does your school have a policy about the number of cases that would necessitate closure of the whole school?	Yes, based on number of cases; please enter number<br>Yes, based on fixed number of cases; please specify<br>No<br>Do not know<br>Prefer not to say	Ask all	Response options are slightly different for SIS Extended (head teacher) as there is only a single 'Yes' response.
SIS Follow-up (head teacher)	Have any of the following sources explained how the school will be notified of confirmed Covid-19 cases among staff?	Department for Education<br>Public Health England<br>Local authority<br>Other, please specify<br>No information received<br>Do not know<br>Prefer not to say	Ask all	Multiple response options allowed.

Source	Question	Response options	Routing	Notes
SIS Baseline (head teacher)	Have you been given guidance about whether your school will be notified about confirmed cases among students from any of the following sources?	Department for Education<br>Public Health England<br>Local authority<br>Other, please specify<br>No information received<br>Do not know<br>Prefer not to say	Ask all	Multiple response options allowed.
SIS Baseline (head teacher);	Have you been given guidance about whether your school will be notified about confirmed cases among household members of students from any of the following sources?	Department for Education<br>Public Health England<br>Local authority<br>Other, please specify<br>No information received<br>Do not know<br>Prefer not to say	Ask all	Multiple response options allowed.
SIS Follow-up (head teacher)	Have you been directly informed by any government or public health agency about confirmed COVID-19 cases in any of the following groups at your school?	Yes, staff case(s)<br>Yes, student case(s)<br>Yes, household member(s) of student(s)<br>No, we have not received any information directly from public health agencies about confirmed cases at our school<br>Do not know<br>Prefer not to say	Ask all	Multiple response options allowed.
SIS Baseline (head teacher);	Has your school so far used the DfE Helpline for advice on COVID-19?	Yes<br>No<br>Not sure<br>Prefer not to say	Ask all	

Follow-up Questionnaire

- **parent/guardian**
- **student over 16 years**
- **staff**

Source	**Question**	**Response options**	Routing	**Notes**
SIS Follow-up (parent/guardian);	In the period between the last school testing visit and this most recent school testing visit, have you/has your child been tested for COVID-19 outside of the school study (this can be a swab, saliva or blood test)?	Yes<br>No<br>Don't know<br>Prefer not to say	Ask all	
SIS Follow-up (over 16 student);	Why did you/your child get tested for COVID-19? (If your child has had more than one test during this period, please select all that apply)	I/my child had symptoms<br>I/my child had contact with someone else with confirmed COVID-19<br>Offered test as part of mass testing of students / community<br>Other	Ask if had a COVID-19 test	Multiple response options allowed.
SIS Follow-up (staff)	Did this test / any of these test result(s) come back positive for COVID-19?	Yes<br>No<br>Don't know	Ask if had a COVID-19 test	
SIS Follow-up (parent/guardian);	What type of test was it? (If you/your child had more than one test outside of the school study during this period, please answer for the first test that came back positive)	Nose / throat swab test (e.g. test for current infection)<br>Blood test (e.g. blood test for antibodies / past infection)<br>Saliva test (spat into cup / tube)<br>Don't know	Ask if tested positive for COVID-19	
SIS Follow-up (over 16 student);	What was the date of this first positive test? (If you cannot remember the exact date, please give an estimate)	[enter date]	Ask if tested positive for COVID-19	Date entered in DD/MM/YYYY format.<br>Only 2020 and 2021 year options available.
SIS Follow-up (staff)	In the past four weeks, have you been aware of anyone testing positive for COVID-19 among people you/your child may have been in contact with?	Yes<br>No<br>Don't know<br>Prefer not to say	Ask all	
SIS Follow-up (parent/guardian);	Please select the option which best describes these contacts: (Select all that apply if your child has been in contact with more than one person who has tested positive)	A household member<br>Someone at school (student or staff)<br>Parent / household member of someone at school<br>Friend or family friend from outside of school<br>Family / relative living in a different household<br>Other (please specify)	Ask if in contact with positive case	

Source	**Question**	**Response options**	Routing	**Notes**
SIS Follow-up (over 16 student);	Please look at the following list of symptoms. Could you tell us whether you/your child experienced any of these symptoms in the week before or after this round of school survey testing.<br><br>1. Fever / chills / high temperature / hot to touch<br>2. New continuous cough<br>3. Loss or change to their sense of smell or taste<br>4. Shortness of breath / difficulty breathing<br>5. Feeling extra tired / Fatigue<br>6. Sore throat<br>7. Blocked or runny nose, sneezing<br>8. Muscle ache / joint pain<br>9. Headaches<br>10. Nausea / vomiting<br>11. Tummy / abdominal pain<br>12. Diarrhoea	Yes<br>No	Ask all	Response required for each symptom
SIS Follow-up (staff)	When did these symptoms first start? (If you cannot remember the exact date, please give an estimate)	[enter date]	Ask if had any symptoms	Date entered in DD/MM/YYYY format, with the year pre-filled as 2021.<br><br>Date required for each symptom selected 'Yes' for.
SIS Follow-up (parent/guardian);	Have you/has your child had a flu vaccination this winter (2020/2021)?	Yes<br>No<br>Don't know<br>Prefer not to say	Ask all	Prefer not to say' is not a response option for SIS Follow-up (over 16 student).
SIS Follow-up (over 16 student);	Have you/has your child had a vaccination for COVID-19?	Yes<br>No<br>Don't know<br>Prefer not to say	Ask all	Prefer not to say' is not a response option for SIS Follow-up (over 16 student).
SIS Follow-up (staff)	If an approved COVID-19 vaccine was offered to your child, would you want your child to have the vaccine? (parent/guardian)<br><br>or<br><br>If an approved COVID-19 vaccine was offered to you, would you choose to have the vaccine? (staff/student)	Yes definitely<br>Unsure but leaning towards yes<br>Unsure but leaning towards no<br>Definitely not	Ask if has not had a COVID-19 vaccine	Slight wording differences for SIS Vaccine.

Source	Question	Response options	Routing	Notes
SIS Follow-up (parent/guardian);	In the period between the last school testing visit and this most recent school testing visit, has anybody else living in your/your child’s primary household (not including you/your child participating in this study) tested positive for COVID-19?	Yes<br>No<br>Don't know<br>Prefer not to say	Ask all	
SIS Follow-up (over 16 student);	If yes, how many people (adults or other children) from your household tested positive for COVID-19 during this period?	[select number of people]	Ask if someone in household tested positive for COVID-19	Response option presented as drop down list from 1 to 10.
SIS Follow-up (staff)	When did they receive a positive test result for COVID-19? (If this household member has had more than one test in this period, please answer for the most recent test; If you do not know the exact date, please give an estimate)	[enter date]	Ask if someone in household tested positive for COVID-19	Date entered in DD/MM/YYYY format.
SIS Follow-up (parent/guardian);	What type of test was it?	Nose / throat swab test (e.g. test for current infection)<br>Blood test (e.g. blood test for antibodies / past infection)<br>Saliva test (spat into cup / tube)<br>Don’t know		
SIS Follow-up (over 16 student);	In the past four weeks, has anyone in the household (not including yourself/your child participating in this study) been asked to self-isolate / stay home because of possible contact with a confirmed case of COVID-19 from outside of your household? (Do not include household members who are isolating because someone else in your household has tested positive)	Yes<br>No<br>Don't know<br>Prefer not to say	Ask for each member of household who tested positive for COVID-19	
SIS Follow-up (staff)	This half term, how many days per week have you usually attended your school? (over 16 student)<br><br>or<br><br>This half term, how many days per week have you worked in-person at school? (staff)	[select number of days]	Ask if someone in household tested positive for COVID-19	Response option presented as drop down list from 0 to 7.
SIS Follow-up (parent/guardian);	Over the past four weeks, have you/has your child ever used public transport (public bus, overground train, tube, DLR, tram, school bus) to travel to or from school?	Yes<br>No<br>Don't know		

Source	**Question**	**Response options**	Routing	**Notes**
SIS Follow-up (over 16 student);	Over the past four weeks, how many times per week would you say you/your child has used public transport to go to or from school?<br><br>Please count each journey (to or from school) separately; for example, if they take public transport to AND from school 5 days a week, that counts as 10 times per week).	[select number]	Ask for each member of household who tested positive for COVID-19	Response option presented as drop down list from 1 to 10 with '11 or more' as the last option.
SIS Follow-up (staff)	When travelling to and from school, which of the following apply?<br><br>1. You/your child wears a face mask or face covering during trips on public transport<br><br>2. You/your child is able to socially distance from others during trips on public transport	1. Always<br>2. Sometimes<br>3. Rarely<br>4. Never<br>5. Prefer not to say<br>6. Don't know	Ask all	Response required for each protective measure.
SIS Follow-up (parent/guardian);	On the most recent day you/your child was at school, approximately how many of the following people did you/they have a conversation with? By conversation, we mean talking to each other one-to-one or in a small group (not speaking to the whole class).<br><br>1. Students in your/their class or same year group<br><br>2. Students not in you/their year group<br><br>3. If '1 to 5', '6 to 10', '11' or more to question above: Of these, how many were in a year group next to yours/theirs (immediately below or above)?<br><br>4. Other people at school (including teachers and staff)<br><br>5. Other people outside school (not including your household)	None<br>1 to 5<br>6 to 10<br>11 or more<br>Don't know<br>Prefer not to say	Ask all	Response required for each social group child had contact.
SIS Follow-up (over 16 student);	While at school over the past 4 weeks, which of the following applied?<br><br>1. You wore a face mask or face covering in indoor school spaces other than classrooms<br><br>2. You used hand sanitiser regularly while in school<br><br>3. You washed your hands with soap regularly<br><br>4. You're socially distanced from students in other bubbles	1. Always<br>2. Sometimes<br>3. Rarely<br>4. Never<br>5. Not applicable<br>6. Prefer not to say	Ask all	Response required for each protective measure.

Source	**Question**	**Response options**	Routing	**Notes**
SIS Follow-up (staff)	While at school, which of the following applied?<br>1. You have worn a face mask or face covering out of class when mixing predominantly with pupils e.g. corridors, playground, sports / music / art activities<br>2. You have worn a face mask out of class when mixing predominantly with other staff e.g. in the staff room / dining room<br>3. You have used hand sanitiser while in school, between classes<br>4. You have used hand sanitiser while in school, during classes<br>5. You have washed your hands with soap regularly<br>6. You have socially distanced from pupils<br>7. You have socially distanced from other staff members	1. Always<br>2. Sometimes<br>3. Rarely<br>4. Never<br>5. Not applicable<br>6. Prefer not to say	Ask if used public transport	Response required for each protective measure.
SIS Follow-up (parent/guardian);	On the most recent day you were at school, approximately how many of the following people did you have a conversation with? By conversation, we mean talking to each other one-to-one or in a small group. (Do not include the pupils you speak to as a whole class; or any members of your own household who works or is a pupil at your school.)<br>1. Pupils from your school<br>2. Staff members from your school<br>3. Other people outside school (not including your household)	None<br>1 to 5<br>6 to 10<br>11 or more<br>Prefer not to say	Ask if used public transport	Response required for each group of people.
SIS Follow-up (over 16 student);	In the past four weeks, have you/has your child taken part in any of the following indoor extra-curricular activities (outside of school) that have involved in-person interaction or contact with people outside your household?<br>1. Youth clubs, scouts, girl guides, music, art or other organised activities<br>2. Voluntary or community work<br>3. Sport, dance<br>4. Tutorials (e.g. for school subjects) or religious classes<br>5. Other	Yes<br>No<br>Don't know	Ask all	Response required for each extra-curricula activity child took part in.

Source	**Question**	**Response options**	Routing	**Notes**
SIS Follow-up (staff)	Thinking of the past four weeks, apart from the usual extra-curricular activities asked just now, did you/your child have indoor contact with anyone from outside your household on a regular (at least once a week) basis?	Yes<br>No<br>Prefer not to say	Ask all	
SIS Follow-up (parent/guardian);	Is that (select all that apply):	Child's other parent / caregiver (e.g. if child lives part of week in other household)<br>A school-friend from same class / year-group<br>A school-friend from different year-group<br>A friend from another school<br>Grandparents<br>A cleaner<br>Childminder<br>Other adults (friends / family)<br>Other (please specify)	Ask all	
SIS Follow-up (over 16 student);	Have you/has your child visited any of the following places at any time in the past four weeks?<br>1. Different household (e.g. a friend's or relative's house)<br>2. Grocery store / supermarkets / pharmacies<br>3. Cinema / Theatre / Concert / Music venue / Shopping centre<br>4. Restaurant / Café / Pub / Food outlet – indoor<br>5. Place of worship (church, mosque, temple, synagogue, etc.)<br>6. Indoor leisure centre / sports centre / gym / swimming pool<br>7. Outdoor recreation area (parks, playing fields, water sports, outdoor pool, etc.) with people outside your household<br>8. Hairdresser / Beauty Salon / Tanning studio<br>9. Used public transport (bus, tube, train, etc.)<br>10. Travelled on a plane<br>11. Attended a party (including weddings and christenings, etc.)<br>12. Other public place (museum, art gallery, theme park, etc.)	Yes<br>No<br>Don't know	Ask all	Response required for each place visited.
SIS Follow-up (staff)	In the past four weeks, have you/has your child been absent from school at all (including times when you/they or your/their class have been sent home to isolate)?	Yes<br>No<br>Don't know	Ask all	

Source	**Question**	**Response options**	Routing	**Notes**
SIS Follow-up (parent/guardian);	During this four week period, how many days of school did you/your child miss in total? (Do not include weekends, inset days or school holidays from this total; count any part-day missed as a whole day of absence)	[select number of days]	Ask all	Response option presented as drop down list from 1 to 20.
SIS Follow-up (over 16 student);	Please tell us whether any of these absences were COVID related (for example you were/child was ill with COVID-like symptoms, you/child tested positive, you were/child was sent home to isolate, etc).	COVID related<br>Not COVID related<br>Both	Ask if child had indoor contact	
SIS Follow-up (staff)	Please select COVID related reasons for absence. (Select all that apply).	1. You/my child tested positive for COVID-19<br>2. You/my child had COVID-19 symptoms / suspected COVID-19 (but no positive test)<br>3. You were/my child was told to isolate due to a potential contact with a case of COVID-19 at school<br>4. You were/my child was isolating due to a potential contact with a case of COVID-19 from outside of school<br>5. School shut for COVID-19 related reasons (e.g. outbreak at school, local lockdown, other)<br>6. Quarantining due to travel outside of the UK<br>7. (Child) shielding<br>8. You have/my child had long-term symptoms after being positive for COVID-19 in the past<br>9. Other COVID-19 related reason (please specify)	Ask all	
SIS Follow-up (parent/guardian);	Please select non-COVID related reasons for absence. (Select all that apply).	1. Non-COVID related respiratory illness (e.g. cold, flu, asthma, croup, bronchiolitis)<br>2. Non-COVID related vomiting / diarrhoea / nausea / abdominal pain<br>3. Other non-COVID related illness<br>4. Medical / dental / hospital appointment / care<br>5. Other non-COVID related reason (please specify)	Ask all	
SIS Follow-up (staff);	Are there any times in the day when students in your bubble might mix with other bubbles or groups? (Select all that apply)	1. Yes, during break<br>2. Yes, during lunch<br>3. Yes, during sports<br>4. Yes, after-school clubs<br>5. Yes, hanging out immediately after school<br>6. No<br>7. Other<br>8. Don’t know<br>9. Prefer not to say	Ask if absent from school	Question wording slightly different for SIS Follow-up (staff).

Source	**Question**	**Response options**	Routing	**Notes**
SIS Vaccine (parent);	In your experience, how well are the following preventive measures being followed?<br>1. Staff wear facemasks or face coverings in the classroom<br>2. Staff wear facemasks or face coverings in corridors or communal areas<br>3. Staff wear facemasks or face coverings in staff rooms or shared office space<br>4. Staff maintain 2m distance from students<br>5. Staff maintain 2m distance from other adults at school<br>6. Staff regularly wash or sanitise hands	Always<br>Sometimes<br>Rarely<br>Never<br>Not applicable<br>Don’t know	Ask if absent from school	Response required for each protective measure.
SIS Vaccine (staff/student)	In your experience, how well are the following preventive measures being followed?<br>1. Students wear facemasks or face coverings in the classroom<br>2. Students regularly wash or sanitise hands<br>3. Students catch coughs or sneezes with tissue or arm	Always<br>Sometimes<br>Rarely<br>Never<br>Not applicable<br>Don’t know	Ask if absence 'COVID related' or 'Both'	Response required for each protective measure.
SIS Follow-up (parent/guardian);	In your experience, how well are the following preventive measures being followed?<br>1. Seats and desks are spaced apart to maintain distance between students<br>2. All desks face forward<br>3. Increased cleaning of frequently touched surfaces<br>4. Windows are kept open / opened at regular intervals to air the classrooms<br>5. Students do not share equipment or learning materials in classrooms<br>6. Students do not carry equipment or learning materials between home and school	Always<br>Sometimes<br>Rarely<br>Never<br>Not applicable<br>Don’t know	Ask if absence 'Not COVID related' or 'Both'	Response required for each protective measure.

Source	**Question**	**Response options**	Routing	**Notes**
SIS Follow-up (over 16 student);	We want to understand the impact of school closures and re-opening on staff wellbeing. Below are some statements of job-related feelings. Please read each statement carefully and decide if you ever feel this way about your job.<br>1. I feel emotionally drained by my work<br>2. I feel used up at the end of the workday<br>3. I feel fatigued when I get up in the morning and have to face another day on the job<br>4. I can easily understand how my students feel about things<br>5. I feel I treat some students as if they were impersonal objects.<br>6. Working with people all day is really a strain for me<br>7. I deal very effectively with the problems of my students<br>8. I feel burned out from my work<br>9. I feel I'm positively influencing other people's lives through my work<br>10. I've become more callous toward people since I took this job<br>11. I worry that this job is hardening me emotionally<br>12. I feel very energetic<br>13. I feel frustrated by my job<br>14. I feel I'm working too hard in my job<br>15. I don't really care what happens to some students<br>16. Working directly with people puts too much stress on me<br>17. I can easily create a relaxed atmosphere with my students<br>18. I feel exhilarated after working closely with my students<br>19. I have accomplished many worthwhile things in this job<br>20. I feel like I'm at the end of my tether<br>21. In my work, I deal with emotional problems very calmly<br>22. I feel students blame me for some of their problems	0 – Never<br>1 – A few times a year or less<br>2 – Once a month or less<br>3 – A few times a month<br>4 – Once a week<br>5 – A few times a week<br>6 – Everyday	Ask all	Response option not mandatory.<br><br>Question wording slightly different for SIS Follow-up (staff), including further elaboration on the response options.

Vaccine Questionnaire

Source	Question	Response options	Routing	Notes
SIS Vaccine (parent);<br>SIS Vaccine (staff/student)	Including you/your child, how many people in total currently live in your household?	[enter number of people]	Ask all	
SIS Vaccine (parent);<br>SIS Vaccine (staff/student)	How many doses have you/has your child received to date?	1 dose<br>2 dose<br>Prefer not to say	Ask if received a COVID-19 vaccination	
SIS Vaccine (parent);<br>SIS Vaccine (staff/student)	When did you/your child receive the most recent vaccination dose?	[enter date]	Ask if received a COVID-19 vaccination	Date entered in DD/MM/YYYY format.
SIS Vaccine (parent);<br>SIS Vaccine (staff/student)	Do you know the name of the COVID-19 vaccine that you/your child was given at the most recent vaccination?	Oxford / AstraZeneca<br>Pfizer / BioNTech<br>Moderna<br>Novavax<br>Janssen / Johnson & Johnson<br>Other<br>Don't know<br>Prefer not to say	Ask if received a COVID-19 vaccination	
SIS Vaccine (parent);<br>SIS Vaccine (staff/student)	Please specify the name of the COVID-19 vaccine you/your child received	[enter name]	Ask if 'Other' COVID-19 vaccine name selected	
SIS Vaccine (parent);<br>SIS Vaccine (staff/student)	Have any member(s) of your household, other than you/your child, received the COVID-19 vaccination to date?	Yes<br>No<br>Don't know<br>Prefer not to say	Ask all	
SIS Vaccine (parent);<br>SIS Vaccine (staff/student)	How many members of your household other than your child have received at least one dose of COVID-19 vaccine?	[enter number of people]	Ask if other members of household have had COVID-19 vaccine	Response option selected from drop down list from 1 to 10.

Source	Question	Response options	Routing	Notes
SIS Follow-up (parent/guardian);<br>SIS Follow-up (staff);<br>SIS Vaccine (parent);<br>SIS Vaccine (staff/student)	If an approved COVID-19 vaccine was offered to your child, would you want your child to have the vaccine? (parent/guardian)<br>or<br>If an approved COVID-19 vaccine was offered to you, would you choose to have the vaccine? (staff/student)	Yes definitely<br>Unsure but leaning towards yes<br>Unsure but leaning towards no<br>Definitely not	Ask if has not had a COVID-19 vaccine	Slight wording differences for SIS Vaccine.
SIS Vaccine (parent);<br>SIS Vaccine (staff/student)	Can you tell us why?	[enter free text answer]	Ask if any 'No' response option to being offered COVID-19 vaccine	
SIS Vaccine (parent);<br>SIS Vaccine (staff/student)	In preparation for, or since the schools re-opened to all students on 8th March 2021, have you/has your child been tested for COVID-19 as part of the mass testing programme at their school (with 'lateral flow test kits')? (Select all that apply)	Yes, my child has been tested on site at school at least once<br>Yes, my child was tested at home using the lateral flow test kit at least once<br>No, my child was never offered any COVID-19 test as part of the school testing programme<br>No, my child was offered, but they did not do a COVID-19 test as part of the school testing programme<br>Prefer not to say		
SIS Vaccine (parent);<br>SIS Vaccine (staff/student)	In preparation for, or since the schools re-opened to all students on 8th March 2021, have you/has your child ever had a positive COVID-19 test result when they were tested using lateral flow kits as part of the school testing programme?	Yes<br>No<br>Prefer not to say	Ask if tested for COVID-19 using later flow test kit	
SIS Vaccine (parent);<br>SIS Vaccine (staff/student)	On what date did you/your child take their most recent lateral flow test (as part of the school testing programme)?	[enter date]	Ask if tested for COVID-19 using later flow test kit	Date entered in DD/MM/YYYY format.
SIS Vaccine (parent);<br>SIS Vaccine (staff/student)	What was the result of your/their test on that date?	Positive<br>Negative<br>Inconclusive<br>Awaiting test result<br>Prefer not to say	Ask if tested for COVID-19 using later flow test kit	

Source	Question	Response options	Routing	Notes
SIS Vaccine (parent);<br>SIS Vaccine (staff/student)	Were you/was your child offered a PCR test to confirm the result of the lateral flow test?	Yes, PCR test was offered and taken<br>Yes, PCR test was offered, but not taken<br>No, PCR test was not offered<br>Prefer not to say	Ask if tested positive for COVID-19 using later flow test kit	
SIS Vaccine (parent);<br>SIS Vaccine (staff/student)	Finally, we would like to understand the use of facemasks in school<br><br>Do you/does your child wear a facemask or face covering in class?	Always<br>Sometimes<br>Rarely<br>Never<br>Prefer not to say	Ask all	
SIS Vaccine (parent);<br>SIS Vaccine (staff/student)	If not, why not?	Exempt because of health condition<br>Not needed because spacing is adequate in class<br>Not required by the school<br>Other<br>Prefer not to say	Ask if never wear a facemask	
SIS Vaccine (parent);<br>SIS Vaccine (staff/student)	Please specify why you/your child does not wear a facemask or face covering in class.	[enter reason]	Ask if select 'Other' reason for not wearing facemask	Free text response.

End of Survey Questionnaire

- **parent/guardian**
- **student over 16 years**
- **staff**

Source	Question	Response options	Routing	Notes
SIS End of Study (staff)	On a scale of 0 - 10, how committed would you say you are to keeping the school open at this time?	1 - not at all committed<br>...<br>10 - extremely committed<br>Prefer not to say	Ask all	Response option selected from drop down list from 0 to 10.
SIS End of Study (parent);<br>SIS End of Study (staff);<br>SIS End of Study (over 16 student)	On a scale of 0 - 10, in your opinion, how much does your child feel they are at risk from COVID-19 infection? (parent)<br>or<br>On a scale of 0-10, how much do you personally feel at risk from COVID-19 infection given your profession? (staff/student)	0 - very low risk<br>...<br>10 - very high risk<br>Prefer not to say	Ask all	Response option selected from drop down list from 0 to 10.
SIS End of Study (parent);<br>SIS End of Study (staff);<br>SIS End of Study (over 16 student)	On a scale of 0 - 10, how safe do/does you/your child feel in school at the moment?	0 - not safe at all<br>...<br>10 - extremely safe<br>Prefer not to say	Ask all	Response option selected from drop down list from 0 to 10.
SIS End of Study (parent);<br>SIS End of Study (staff);<br>SIS End of Study (over 16 student)	On a scale of 0 - 10, how well would you say your/your child's school has dealt with implementing preventive measures at school?	0 - not very well<br>...<br>10 - extremely well<br>Prefer not to say	Ask all	Response option selected from drop down list from 0 to 10.

Source	Question	Response options	Routing	Notes
SIS End of Study (parent)	We want to understand the impact of school closures and reopening on young people's/children's mental wellbeing and behaviour.<br><br>Which of these phrases best describes you/your child? For each item, please mark the box for 'Not True', 'Somewhat True' or 'Certainly True'.<br><br>It would help us if you answered all items as best you can even if you are not absolutely certain or the item seems daft! Please give your answers on the basis of your child's behaviour over the last six months.<br><br>If you prefer not to answer these questions you can leave them blank and move to the next section.<br><br>1. Considerate of other people's feelings<br>2. Restless, overactive, cannot stay still for long<br>3. Often complains of headaches, stomach aches, or sickness<br>4. Shares readily with other children (treats, toys, pencils etc)<br>5. Often has temper tantrums or hot tempers<br>6. Rather solitary, tends to play alone<br>7. Generally obedient, does what adults request<br>8. Many worries, often seems worried<br>9. Helpful if someone is hurt, upset or feeling ill<br>10. Constantly fidgeting or squirming<br>11. Has at least one good friend<br>12. Often fights with other children or bullies them<br>13. Often unhappy, down-hearted or tearful<br>14. Generally liked by other children<br>14. Easily distracted, concentration wanders<br>16. Nervous or clingy in new situations, easily loses confidence<br>17. Kind to younger children<br>18. Often lies or cheats<br>19. Picked on or bullied by other children<br>20. Often volunteers to help others (parents, teachers, other children)<br>21. Thinks things out before acting<br>22. Steals from home, school or elsewhere<br>23. Gets on better with adults than other children<br>24. Many fears, easily scared<br>25. Sees tasks through to the end, good attention span	Not true<br>Somewhat true<br>Certainly true	Ask all	Response required for each phrase, but this is an optional question.<br><br>If the respondent prefers not to answer, this can be left blank.

Source	Question	Response options	Routing	Notes
SIS End of Study (over 16 student)	We want to understand the impact of school closures and reopening on children's mental wellbeing and behaviour.<br><br>Which of these phrases best describes your child? For each item, please mark the box for 'Not True', 'Somewhat True' or 'Certainly True'.<br><br>It would help us if you answered all items as best you can even if you are not absolutely certain or the item seems daft! Please give your answers on the basis of your child's behaviour over the last six months.<br><br>If you prefer not to answer these questions you can leave them blank and move to the next section.<br><br>1. I try to be nice to other people. I care about their feelings<br>2. I am restless, I cannot stay still for long<br>3. I get a lot of headaches, stomach-aches or sickness<br>4. I usually share with others (food, games, pens etc.)<br>5. I get very angry and often lose my temper<br>6. I am usually on my own. I generally play alone or keep to myself<br>7. I usually do as I am told<br>8. I worry a lot<br>9. I am helpful if someone is hurt, upset or feeling ill<br>10. I am constantly fidgeting or squirming<br>11. I have one good friend or more<br>12. I fight a lot. I can make other people do what I want<br>13. I am often unhappy, down-hearted or tearful<br>14. Other people my age generally like me<br>14. I am easily distracted, I find it difficult to concentrate<br>16. I am nervous in new situations. I easily lose confidence<br>17. I am kind to younger children<br>18. I am often accused of lying or cheating<br>19. Other children or young people pick on me or bully me<br>20. I often volunteer to help others (parents, teachers, children)<br>21. I think before I do things<br>22. I take things that are not mine from home, school or elsewhere<br>23. I get on better with adults than with people my own age<br>24. I have many fears, I am easily scared<br>25. I finish the work I'm doing. My attention is good	Not true<br>Somewhat true<br>Certainly true	Ask all	

Source	Question	Response options	Routing	Notes
SIS End of Study (parent);<br>SIS End of Study (staff);<br>SIS End of Study (over 16 student)	We would now like to ask about your/your child's usual use of health care services before the COVID-19 pandemic. Please think about January to December 2019; before the COVID-19 pandemic.<br><br>In 2019, how many times were/was you/your child admitted to hospital for at least one night?	None<br>Once<br>Twice<br>Three times or more<br>Prefer not to say<br>Don't know	Ask all	
SIS End of Study (parent);<br>SIS End of Study (staff);<br>SIS End of Study (over 16 student)	In 2019, how many times did you/your child have an Outpatient Department appointment?	None<br>Once<br>Twice<br>Three times or more<br>Prefer not to say<br>Don't know	Ask all	
SIS End of Study (parent);<br>SIS End of Study (staff);<br>SIS End of Study (over 16 student)	In 2019, approximately how many times did you speak with your GP about your child's health (by phone or in person)? (parent)<br><br>or<br><br>In 2019, approximately how many times did you/your parent or carer speak with your GP (by phone or in person)? (staff/student)	None<br>Once<br>Twice<br>Three times or more<br>Prefer not to say<br>Don't know	Ask all	
SIS End of Study (parent);<br>SIS End of Study (staff);<br>SIS End of Study (over 16 student)	We would now like to ask you some questions about any experiences your child has had of COVID-19 infection and ongoing symptoms that have lasted more than four weeks afterwards.<br><br>Do you think you/your child have/has, or have/has previously had, COVID-19?	Yes, diagnostic confirmed with a lab test<br>Yes, suspected diagnostic (by GP or NHS 111)<br>Yes, on basis of lateral flow test (result in 30 minutes)<br>Yes, suspected due to symptoms - no test/medical diagnosis<br>No<br>Prefer not to say<br>Don't know	Ask all	
SIS End of Study (parent);<br>SIS End of Study (staff);<br>SIS End of Study (over 16 student)	Were/was or are/is you/your child experiencing ongoing symptoms more than four weeks since the start of their COVID-19 infection, that are not explained by something else?<br><br>If you/your child first had COVID-19 less than 4 weeks ago please respond 'No' to this question.	Yes<br>No<br>Prefer not to say<br>Don't know	Ask if think you/your child has had COVID-19	

Source	Question	Response options	Routing	Notes
SIS End of Study (parent);<br>SIS End of Study (staff);<br>SIS End of Study (over 16 student)	Did or does this reduce your/their ability to carry-out day-to-day activities compared with the time before you/they had COVID-19?	Yes, a little<br>Yes, a lot<br>Not at all<br>Prefer not to say<br>Don't know	Ask if think you/your child has had COVID-19	
SIS End of Study (parent);<br>SIS End of Study (staff);<br>SIS End of Study (over 16 student)	Which, if any, of the following symptoms are, or were, ongoing more than 4 weeks since the start of your/their COVID-19 infection? For each one, please select whether it was or has been experienced only since the COVID-19 infection, if it was pre-existing but made worse, or if it has not been experienced since the COVID-19 infection.<br><br>1. Fever<br>2. Headache<br>3. Muscle ache<br>4. Weakness / tiredness<br>5. Nausea / vomiting<br>6. Abdominal pain<br>7. Diarrhoea<br>8. Loss of appetite<br>9. Loss of taste<br>10. Loss of smell<br>11. Sore throat<br>12. Cough<br>13. Shortness of breath<br>14. Chest pain<br>15. Palpitations<br>16. Vertigo / dizziness<br>17. Worry / anxiety<br>18. Low mood / not enjoying anything<br>19. Trouble sleeping<br>20. Memory loss or confusion<br>21. Difficulty concentrating<br>22. Sensations in skin, such as prickling, tingling or burning<br>23. Other	Only since COVID-19 infection<br>Pre-existing but made worse<br>Not experienced since initial COVID-19 infection<br>Prefer not to say<br>Don't know	Ask if think you/your child has had COVID-19	Response required for each symptom and this is a mandatory question.<br><br>If the respondent prefers not to answer, this can be left blank.<br><br>For 'Other' symptom, respondents are asked to please specify in a follow up question if 'Only since COVID-19 infection' or 'Pre-existing but made worse' is selected.

Source	Question	Response options	Routing	Notes
SIS End of Study (parent);<br>SIS End of Study (staff);<br>SIS End of Study (over 16 student)	How long did the ongoing symptoms continue, or have they continued, since the start of your/their COVID-19 infection?	4 to 12 weeks<br>13 weeks to 6 months<br>Over 6 months<br>Prefer not to say<br>Don't know	Ask if think you/your child has had COVID-19	
SIS End of Study (parent);<br>SIS End of Study (staff);<br>SIS End of Study (over 16 student)	Have you accessed, or did you access, any medical help for the ongoing symptoms/for your child for the ongoing symptoms, from any of the following? Select all that apply (staff/student)<br><br>or<br><br>Have you accessed, or did you access, any medical help for your child for the ongoing symptoms, from any of the following? Select all that apply (parent)	Contacted NHS 111, by phone or online<br>Visited pharmacist<br>Consulted GP or practice nurse over the phone or online<br>Consulted GP or practice nurse face to face<br>Walk-in centre<br>Hospital Outpatient Department<br>Accident and emergency (A&E)<br>Hospital admission<br>Other<br>No, did not seek medical attention	Ask if think you/your child has had COVID-19	This is an optional question, but respondents can select all that apply.<br><br>For 'Other' medical help selected, respondents are asked to please specify in follow up question.
SIS End of Study (parent);<br>SIS End of Study (staff);<br>SIS End of Study (over 16 student)	Overall, which of the following options best describes if or how your/your child's ongoing symptoms (those lasting for more than 4 weeks since the start of their COVID-19 infection) have changed over time?	Got much worse<br>Got somewhat worse<br>Stayed the same<br>Somewhat improved<br>Greatly improved<br>Got completely better / cleared up<br>Prefer not to say	Ask if think you/your child has had COVID-19	
SIS End of Study (parent);<br>SIS End of Study (staff);<br>SIS End of Study (over 16 student)	Which of these options best describes your/their experience of the ongoing symptoms? Select all that apply	Seriousness of any symptom(s) (has) varied up and down<br>The types of symptoms experienced (have) changed<br>Symptoms (have) stayed the same<br>Don't know	Ask if think you/your child has had COVID-19	This is an optional question, but respondents can select all that apply.

Source	Question	Response options	Routing	Notes
SIS End of Study (parent);<br>SIS End of Study (over 16 student)	How much of your/your child’s schooling have you/they attended, including in person and / or by remote learning, while experiencing ongoing symptoms (those lasting for more than 4 weeks since the start of their COVID-19 infection)?<br>Remote learning includes having access to live or recorded lessons online, or to other resources, away from school.	All of it<br>More than half<br>About half<br>Less than half<br>None<br>Prefer not to say<br>Don’t know	Ask if think you/your child has had COVID-19	
SIS End of Study (parent);<br>SIS End of Study (over 16 student)	How much, if at all, has experiencing ongoing symptoms (those lasting for more than 4 weeks since the start of their COVID-19 infection) affected your/your child’s education?	A lot, for the worse<br>A little, for the worse<br>Not affected<br>A little, for the better<br>A lot, for the better<br>Prefer not to say<br>Don’t know	Ask if think you/your child has had COVID-19	
SIS End of Study (parent);<br>SIS End of Study (over 16 student)	To what extent do you feel that the school has supported you/your child to manage the impact of the ongoing symptoms on your/their education?	They were given all the support needed<br>They were given most of the support needed<br>They were given less support than was needed<br>They were given none of the support needed<br>Not applicable: did not need any support<br>Prefer not to say<br>Don’t know	Ask if think you/your child has had COVID-19	
SIS End of Study (staff)	In total, how many working days have you been absent due to experiencing ongoing symptoms (those lasting for more than 4 weeks since the start of your COVID-19 infection)? By ‘working days’ we mean either at school or remotely (if that is possible in your role).<br>If you cannot remember for certain, please provide an estimate.	No days absent<br>Less than 5 days<br>6-10 days<br>11-20 days<br>21-30 days<br>31-60 days<br>61 days and over<br>Prefer not to say	Ask if think you have had COVID-19	

Source	Question	Response options	Routing	Notes
SIS End of Study (staff)	In total, how many working days have you worked remotely, specifically due to the ongoing symptoms (those lasting for more than 4 weeks since the start of your COVID-19 infection)?<br>If you cannot remember for certain, please provide an estimate.	Not applicable: cannot work remotely in my role<br>No remote working due to ongoing symptoms<br>Less than 5 days<br>6-10 days<br>11-20 days<br>21-30 days<br>31-60 days<br>61 days and over<br>Prefer not to say	Ask if think you have had COVID-19	
SIS End of Study (staff)	To what extent have the ongoing symptoms (those lasting for more than 4 weeks since the start of your COVID-19 infection) reduced your ability to carry out your activities at work?	Ability not affected at all<br>Ability reduced a little<br>Ability reduced by about half<br>Ability reduced a lot<br>Unable to work at all<br>Prefer not to say<br>Don't know	Ask if think you have had COVID-19	
SIS End of Study (staff)	To what extent do you feel the school has supported you in relation to your ongoing symptoms? For example, in allowing time off work, flexible or reduced hours, change to duties or ways of working.	I was given all of the support needed<br>I was given a lot of the support needed<br>I was given only a little of the support needed<br>I was given none of the support needed<br>Not applicable: did not need any support<br>Prefer not to say<br>Don't know	Ask if think you have had COVID-19	

Source	Question	Response options	Routing	Notes
SIS End of Study (parent);<br>SIS End of Study (staff);<br>SIS End of Study (over 16 student)	We would now like to ask you some questions about your experience of taking part in the Schools Infection Survey. Your feedback is important to help us improve the design of studies like this in the future.<br><br>1. The survey questions were easy to understand<br>2. There were too many questions<br>3. Some questions were intrusive<br>4. It took too long to complete the questionnaires<br>5. All of the questions seemed necessary<br>6. The topics covered in the questionnaires were relevant<br>7. I took part in the survey to help fight against COVID-19<br>8. This survey was a waste of my time<br>9. I believed my answers would be confidential<br>10. The questionnaires were easy to navigate<br>11. The frequency of testing for COVID-19 was appropriate	Strongly agree<br>Agree<br>Neither agree nor disagree<br>Disagree<br>Strongly disagree	Ask all	Response required for each statement about the Schools Infection Survey and this is a mandatory question.
SIS End of Study (staff)	Thinking of the finger prick test used for COVID-19 in the study, which did you prefer?	Doing the finger prick test at home myself<br>Doing the finger prick test at school with a nurse<br>No preference<br>Prefer not to say	Ask all	
SIS End of Study (over 16 student)	If it was part of the study would you have been willing to do a finger pricking test (drop of blood from the tip of your finger) for COVID-19?	Yes definitely<br>Unsure but probably yes<br>Unsure but probably no<br>Definitely no<br>Prefer not to say	Ask all	
SIS End of Study (over 16 student)	Did your parent/carer complete the questionnaires for you?	All questions completed for you<br>Some questions completed for you<br>I completed all questions myself	Ask all	
SIS End of Study (staff)	Did you have a child/children of your own at this school?	Yes<br>No<br>Prefer not to say	Ask all	
SIS End of Study (parent);<br>SIS End of Study (staff)	Overall, are you left feeling that taking part in the survey was worthwhile?	Yes<br>No<br>Don’t know	Ask all	
